# Ru(II) Complexes with 3,4-Dimethylphenylhydrazine: Exploring In Vitro Anticancer Activity and Protein Affinities

**DOI:** 10.3390/biom15030350

**Published:** 2025-02-28

**Authors:** Jasmina Dimitrić Marković, Dušan Dimić, Thomas Eichhorn, Dejan Milenković, Aleksandra Pavićević, Dragoslava Đikić, Emilija Živković, Vladan Čokić, Tobias Rüffer, Goran N. Kaluđerović

**Affiliations:** 1 Faculty of Physical Chemistry, University of Belgrade, 11000 Belgrade, Serbiaaleks.pavicevic@ffh.bg.ac.rs (A.P.); 2 Department of Engineering and Natural Sciences, University of Applied Sciences Merseburg, D-06217 Merseburg, Germany; thomas.eichhorn@hs-merseburg.de; 3 Department of Science, Institute for Information Technologies, University of Kragujevac, 34000 Kragujevac, Serbia; 4 Institute for Medical Research, University of Belgrade, 11000 Belgrade, Serbia; dragoslava@imi.bg.ac.rs (D.Đ.); emilija.zivkovic@imi.bg.ac.rs (E.Ž.); vl@imi.bg.ac.rs (V.Č.); 5 Institute of Chemistry, Chemnitz University of Technology, Straße der Nationen 62, D-09111 Chemnitz, Germany; tobias.rueffer@chemie.tu-chemnitz.de

**Keywords:** Ru(II) complexes, HSA, spin-probing, MIA PaCa-2/MDA-MB-231/HS-5 cells, MDA/PC levels, cell cycle

## Abstract

Two new Ru(II) complexes, mononuclear [RuCl_2_(η^6^-*p*-cymene)(3,4-dmph-κ*N*)] (**1**) and the binuclear complex [{RuCl(η^6^-*p*-cymene)}_2_(μ-Cl)(μ-3,4-dmph-κ^2^*N*,*N*′)]Cl (**2**; 3,4-dmph = 3,4-dimethylphenylhydrazine), are synthesized and experimentally and theoretically structurally characterized utilizing ^1^H and ^13^C NMR and FTIR spectroscopy, as well as DFT calculations. Degradation product of **2**, thus ([{RuCl(η^6^-*p*-cymene)}_2_(μ-Cl)(μ-3,4-dmph-κ^2^*N*,*N*′)][RuCl_3_(η^6^-*p*-cymene)] (**2b**) was characterized with SC-XRD. In the crystals of **2b**, the cationic and anionic parts interact through N-H^...^Cl hydrogen bridges. The spectrofluorimetric measurements proved the spontaneity of the binding processes of both complexes and HSA. Spin probing EPR measurements implied that **1** and **2** decreased the amount of bound 16-doxylstearate and implicated their potential to bind to HSA more strongly than the spin probe. The cytotoxicity assessment of both complexes against the MDA-MB-231 and MIA PaCa-2 cancer cell lines demonstrated a clear dose-dependent decrease in cell viability and no effect on healthy HS-5 cells. Determination of the malondialdehyde and protein carbonyl concentrations indicated that new complexes could offer protective antioxidant benefits in specific cancer contexts. Gel electrophoresis measurements showed the reduction in MMP9 activity and indicated the potential of **1** in limiting the cancer cells’ invasion. The annexin V/PI apoptotic assay results showed that **1** and **2** exhibit different selectivity towards MIA PaCa-2 and MDA-MB-231 cancer cells. A comparative molecular docking analysis of protein binding, specifically targeting acetylcholinesterase (ACHE), matrix metalloproteinase-9 (MMP-9), and human serum albumin (HSA), demonstrated distinct binding interactions for each complex.

## 1. Introduction

As the leading cause of mortality worldwide, cancer remains one of the most challenging scientific topics nowadays, necessitating the ongoing search and continuous pursuit of innovative therapeutic strategies with minimal adverse effects and maximal therapeutic efficacy. Besides radiation and surgery, cancer therapy is mainly based on conventional chemotherapeutics, among which cisplatin stands out today as one of the most successful anticancer drugs. Non-selectivity, i.e., toxicity and acquisition of resistance, still represents a huge drawback in the clinical application of cisplatin [1,2,3]. Namely, cisplatin, for the most part through interaction with the DNA molecule leads to the death of tumor cells, but also it affects healthy cells, especially those that have a high proliferative capacity potential or participate in the detoxification of the organism such as the liver and kidney cells [4,5,6,7,8,9,10,11,12,13].

Among other alternatives to cisplatin Ru(II/III) complexes have emerged as promising candidates in anticancer research due to their thermodynamic and kinetic stability under physiological conditions, a wide range of ligands and decorations, and octahedral geometry [14]. They have the ability to interact with biological molecules in ways that can induce cell death in cancer cells. Recent advancements highlight the potential of these complexes to overcome some limitations associated with traditional platinum-based drugs. Ruthenium is a transition metal with versatile oxidation states and coordination geometries which enable fine-tuning of its biological properties due to the possibility of incorporation of different ligands resulting in increased selectivity and potency against cancer cells. Ru(II/III) complexes undergo various mechanisms leading to cancer cell death: binding to DNA and proteins, modulating key signaling pathways involved in cell survival and proliferation, generating reactive oxygen species (ROS), and exhibiting photochemical reactivity that can be exploited for targeted therapy [15,16,17,18,19,20,21,22,23,24,25,26,27,28,29]. Ru(II) complexes typically show higher bioactivity and reactivity [30]. Several ruthenium-based compounds (NAMI-A, NKP-1339, and TLD-1433) entered clinical trials, although with various success [31]. Cationic nucleolipid nanosystem for the nano delivery of a ruthenium(III) complex showed promising results in mouse models validated through the reduction of tumors without animal suffering [32].

This study aims to delve into the specifics of hydrazine derivatives ligands in the context of development of two new, mono- and binuclear Ru(II) complexes, namely dichlorido(η^6^-*p*-cymene)(3,4-dimethylphenylhydrazine-κ*N*)ruthenium(II) (**1**) and chlorido(μ-chlorido(η^6^-*p*-cymene)(μ-3,4-dimethylphenylhydrazine-κ^2^*N*,*N*′)ruthenium(II)-dimer chloride (**2**). As ligands, hydrazine derivatives can effectively coordinate with various metal ions, forming stable complexes that show potential anticancer activity. The structural versatility of hydrazine derivatives allows for modifications that can enhance their selectivity and efficacy against cancer cells while minimizing toxicity to normal tissues. Additionally, the presence of nitrogen-rich functional groups in hydrazine derivatives can facilitate interactions with biological macromolecules, potentially leading to improved drug delivery and targeting efficiency [33,34].

For structural characterization, ^1^H, ^13^C NMR, and FTIR methods are used. DFT calculations further have complemented the investigation by enhancing our understanding of their electronic structure and reactivity. Molecular docking has facilitated the exploration of binding affinities and interaction modes between new complexes and the target proteins, human serum albumin, acetylcholinesterase, and matrix metalloproteinase-9, providing vital information about key interactions between the ligands and the proteins, binding affinity and binding modes. Understanding how new Ru(II) complexes interact with ACHE and MMP-9 is crucial, as both enzymes are instrumental in cancer progression, ACHE through its role in cell signaling and proliferation, and MMP-9 in facilitating tumor invasion and metastasis. Similarly, examining the interaction with HSA is essential for assessing the stability and bioavailability of Ru(II) complexes in therapeutic applications, especially in cancer treatment, and conformational changes in the albumins caused by interaction with complexes which can provide clues about the mechanism of action and the effectiveness of these complexes.

Assessing the cytotoxicity of complexes **1** and **2** is done by employing the MTT test which provides insights into cell viability, and overall cytotoxic effects. In conjunction with this assay, oxidative stress markers, such as malondialdehyde (MDA) and protein carbonyls (PC), have provided vital information regarding the cellular response to oxidative damage induced by the treatment. Examination of the cell cycle provides critical insights into cellular dynamics, growth, and health. By analyzing the distribution of cells across different phases (G0/G1, S, G2/M), it has been possible to assess the proliferation rate and ascertain whether cells are actively dividing or in a quiescent state.

## 2. Materials and Methods

### 2.1. Materials and Instrumentation

Ligand precursor 3,4-dimethylphenylhydrazine hydrochloride (BLDPharm), [{RuCl_2_(η^6^-*p*-cymene)}_2_] (Carbolutions), LiOH × H_2_O (Sigma Aldrich, Darmstadt, Germany), methanol (Carl Roth), diethyl ether (Carl Roth) and 2-propanol (Carl Roth) were obtained commercially and used without further purification. Deuterated solvents, CDCl_3_ and MeOD, were procured from Deuterosolvents GmbH and used for NMR spectroscopy. NMR spectra of the synthesized complexes, including ^1^H (400.23 MHz) and ^13^C NMR (100.1 MHz), were recorded using a Bruker Avance™ 400 MHz Spectrometer. Elemental analysis was performed and the Institute of Chemistry, Chemnitz University of Technology (Heraeus VARIO EL oven). The stability of compounds was examined by UV-VIS spectroscopy on a Thermo Scientific UV-VIS Spectrometer (Thermo Fisher Scientific, Waltham, MA, USA) between 800 and 200 nm. For this experiment, 1 × 10^−2^ M solutions of complexes in DMSO were prepared and further diluted to 1 × 10^−4^ M solutions in PBS. The measurements were repeated for 24 h.

### 2.2. Synthesis

Dichlorido(η^6^-*p*-cymene)(3,4-dimethylphenylhydrazine-κ*N*)ruthenium(II) (**1**)

In a 25 mL flask, [{RuCl_2_(η^6^-*p*-cymene)}_2_] (129 mg, 0.2 mmol) and LiOH × H_2_O (17.1 mg, 0.4 mmol) were suspended in 2-propanol (5 mL) and stirred while degassing with nitrogen at room temperature for 30 min and a deep-red suspension obtained. Then, 3,4-dimethylphenylhydrazine hydrochloride (91 mg, 0.5 mmol) was added under nitrogen. The reaction mixture is turned into an orange suspension overnight. The product was filtered off and washed twice with diethyl ether (2 × 5 mL) and dried in air. Yield 137 mg (74%).

Anal. Calcd for C_18_H_26_Cl_2_N_2_Ru (442.39): C, 48.87; H, 5.92; N, 6.33. Found: C, 48.47; H, 5.76; N, 6.40. ^1^H NMR (400 MHz, CDCl_3_) *δ* 7.02 (d, *J* = 7.9 Hz, C*H*_Ph_, 1H), 6.71 (m, C*H*_Ph_, 2H), 5.54 (d, *J* = 5.8 Hz, C*H*_Cym_, 2H), 5.34 (d, *J* = 5.8 Hz, C*H*_Cym_, 2H), 5.29 (s, N*H*_2_, 2H), 3.02 (hept, *J* = 6.1 Hz, C*H*(CH_3_)_2_, 1H), 2.28 (s, CC*H*_3_, 3H), 2.21 (s, CC*H*_3_, 3H), 2.19 (s, N*H*, 1H), 2.18 (s, CC*H*_3_, 3H), 1.33 (d, *J* = 7.0 Hz, CH(C*H*_3_)_2_, 6H) ppm; ^13^C NMR (100 MHz, CDCl_3_) *δ* 146.6 (*C*N_Ph_), 137.8 (C*C*CHCN_Ph_), 131.2 (*C*CCHCN_Ph_), 130.4 (*C*HCHCN_Ph_), 117.3 (C*C*HCN_Ph_), 113.1 (CH*C*HCN_Ph_), 102.6 (*C*CH(CH_3_)_2_), 97.3 (CH_3_*C*_cym_), 81.8 (C*C*HCH_cym_), 80.6 (C*C*HCH_cym_), 30.9 (*C*H(CH_3_)_2_), 22.4 (CH(*C*H_3_)_2_), 19.9 (C*C*H_3_), 19.0 (C*C*H_3_), 18.7 (C*C*H_3cym_) ppm.

Chlorido(μ-chlorido)(η^6^-*p*-cymene)(μ-3,4-dimethylphenylhydrazine-κ^2^*N*,*N*′)ruthenium(II)-dimer chloride (**2**)

In a 25 mL flask, [{RuCl_2_(η^6^-*p*-cymene)}_2_] (129 mg, 0.2 mmol) and LiOH × H_2_O (8.6 mg, 0.2 mmol) were suspended in methanol (5 mL) and stirred while degassing with nitrogen at room temperature for 30 min and a deep-red suspension was obtained. Then, 3,4-dimethylphenylhydrazine hydrochloride (37.1 mg, 0.21 mmol) was added under nitrogen. The reaction mixture turns into a red solution overnight. Diethyl ether (2 × 10 mL) is added and the flask is stored at 5 °C. The red product was filtered off after ten days washed twice with diethyl ether (2 × 5 mL) and dried in air. Yield 77 mg (51%).

Anal. Calcd for C_28_H_40_Cl_4_N_2_Ru_2_ (748.58): C, 44.93; H, 5.39; N, 3.74. Found: C, 44.74; H, 5.47; N, 3.82. ^1^H NMR (400 MHz, MeOD): *δ* 7.78–7.65 (m, C*H*_Ph_, 1H), 7.36 (d, *J* = 6.9 Hz, C*H*_Ph_, 0.5 H), 7.26 (d, *J* = 7.5 Hz, C*H*_Ph_, 0.5 H), 7.12–7.01 (m, C*H*_Ph_,1H), 5.67–5.62 (m, C*H*_Cym_, 2H), 5.52 (d, *J* = 5.8 Hz, C*H*_Cym_, 2H), 5.50 (d, *J* = 5.9 Hz, C*H*_Cym_, 2H), 5.37, 5.38 (s, N*H_2_*, 2H), 5.25, 5.27 (s, N*H*, 1H), 5.10 (s, C*H*_Cym_, 0.5 H), 4.98 (s, C*H*_Cym_, 0.5 H), 4.73 (d, *J* = 6.0 Hz, C*H*_Cym_, 1H), 2.86 (h, *J* = 6.9 Hz, C*H*(CH_3_)_2_, 1H), 2.73 (m, C*H*(CH_3_)_2_, 1H), 2.36 (s, CC*H*_3_, 3H), 2.34 (s, CC*H*_3_, 3H), 2.13 (s, CC*H*_3_, 3H), 2.01 (s, CC*H*_3_, 3H) 1.31 (d, *J* = 6.9 Hz, CH(C*H*_3_)_2_, 3H), 1.28 (d, *J* = 7.0 Hz, CH(C*H*_3_)_2_, 3H), 1.27–1.24 (m, CH(C*H*_3_)_2_, 3H), 1.11–1.10 (m, CH(C*H*_3_)_2_, 3H); ^13^C NMR (100 MHz, MeOD): *δ* 145.7 (*C*N_Ph_), 133.2 (C*C*CHCN_Ph_), 128.7 (*C*_Ph_), 127.8 (*C*_Ph_), 126.9 (*C*_Ph_), 126.3 (*C*_Ph_), 125.1 (*C*_Ph_), 123.3 (*C*_Ph_), 119.4 (C*C*HCN_Ph_), 119.2 (C*C*HCN_Ph_), 105.5 (*C*CH(CH_3_)_2_), 105.0 (*C*CH(CH_3_)_2_), 96.4 (CH_3_*C*_cym_), 95.4 (CH_3_*C*_cym_), 83.9, 82.6, 80.1, 79.2, 76.6 (5 × C*C*HCH_cym_), 30.0 (*C*H(CH_3_)_2_), 29.8 (*C*H(CH_3_)_2_), 20.1 (C*C*H_3_), 19.1 (C*C*H_3_), 16.5 (C*C*H_3_), 16.3 (C*C*H_3_) ppm.

### 2.3. SC-XRD of the [{RuCl(η^6^-p-cymene)}_2_(μ-Cl)(μ-3,4-dmph-κ^2^N,N′)][RuCl_3_(η^6^-p-cymene)] (***2b***)

The compound **2b** was prepared during attempts to crystallize a cationic binuclear Ru(II) complex **2** from a chloroform solution. Upon slow evaporation of the solvent at room temperature, crystals suitable for X-ray diffraction were obtained. SC-XRD data for **2b** were collected on a Rigaku Oxford Gemini S diffractometer diffractometer equipped with Mo-Kα radiation (λ = 0.71073 Å). The structure was solved using direct methods (WinGX software) and refined with full-matrix least-squares against F² using SHELXL-2013 (Version 2014.1) [35,36]. The unit cell comprises a chloroform-packing solvent molecule. This molecule was refined as being disordered over two positions with occupation factors of 0.18 and 0.57 for C1C/Cl1c-Cl3c and C1C’/Cl1c’-Cl3c’, respectively. The overall occupancy was set to 0.75. Likely, this model does not reflect the nature of the non-bonding packing solvent almost precisely; however, more complicated models are given in their refinement unreliable results. Moreover, a disorder of the 3,4-dmph was noticed and the carbon atoms C21–C29 were refined to split occupancies of 0.45 and 0.55 for C21′–C29′. The positions of nitrogen-bonded hydrogen atoms were taken from different Fourier maps and refined freely. Supplementary crystallographic data for **2b** have been deposited with the Cambridge Crystallographic Data Centre under reference number 2418541. These data are available free of charge and can be accessed via the CCDC website at https://www.ccdc.cam.ac.uk/data_request/cif, accessed on 23 February 2025.

### 2.4. Theoretical Calculations and QTAIM Analysis

The structures of complex **1** and complex cation (**2**–Cl)^+^ were optimized in the Gaussian 09 Program Package without any geometrical constraints [37]. The B3LYP functional in conjunction with 6-311++G(d,p) basis set for H, C, N, Cl, and def2-TZPV basis set for Ru(II) ions was selected [38,39,40,41]. The absence of imaginary frequencies was taken as proof that the minima on the potential energy surface were found. The intramolecular stabilization interactions between the central metal ion and surrounding donor atoms were examined by the Quantum Theory of Atoms in Molecules (QTAIM) approach, as proposed by Bader [42,43,44]. The values of electron density, Laplacian, and others were obtained for the Bond Critical Points (BCPs) in the AIMAll program package [45]. The structures of complex compounds were optimized in solvents to mimic the chemical environment of the NMR experiment. The selected model was the Conductor-like polarizable continuum (CPCM) model [46]. The ^13^C NMR chemical shifts were calculated by the GIAO method with tetramethylsilane (TMS) as a reference compound [47].

### 2.5. Fluorescence Spectra

Fluorescence spectra of investigated complexes and HSA/BSA solutions were recorded at several temperatures, 30 °C, 33 °C, and 37 °C, on an Agilent spectrofluorometer, model Cary Eclipse. The excitation wavelength was set to 285 nm, while the emission spectrum was recorded in the interval from 315 nm to 600 nm. BSA solution (phosphate buffer pH = 7.6) with a concentration of 5 × 10^−6^ M was titrated (5–10 times) with a complex solutions (5 × 10^−4^ M). Before each addition of complexes **1** and **2** to the HSA solutions, the cuvette was thermostated for 2 min for each tested temperature, and then the fluorescence quenching spectra of proteins were recorded. Recordings started 2 min after adding complex **1**/complex **2** solutions to reach the desired temperature. The data were analyzed according to the double-log Stern-Volmer (SV) equation:

(1) $$\log\left(\frac{I_{0}-I_{Q}}{I_{Q}}\right)=\log K_{b}+n\log\left[Q\right]$$

where K_b_ and n are the binding constants and the number of binding sites per HSA molecule. The binding constants were determined from the intercept values on the SV diagrams and the double log SV equation (Table 1). These values are further used for van’t Hoff plots, lnKb = f(T^−1^), from which the Gibbs free energy, enthalpy, and entropy changes were determined.

### 2.6. Molecular Docking

The inhibitor efficiency of **1** and **2** against ACHE, MP-9, and HSA receptors were examined using the molecular docking simulations. The AutoDock 4.2 software [48] was used to examine the binding affinity of investigated compounds. The pockets and binding sites of the receptor were determined by the AutoGridFR (AGFR) program [49]. The crystal structures of examined receptors (PDB IDs: 7p1p [50], 4or0 [51], and 6esm [52] were extracted from the RCSB Protein Data Bank in PDB format. The target receptors were prepared for docking by removing the co-crystallized ligand, water molecules, and cofactors. For this purpose, BIOVIA Discovery Studio 4.0 [53] was employed. The AutoDockTools (ADT) [48] graphical user interface was used to calculate the Kollman partial charges and to add polar hydrogen. The Ru initial autodock parameters were set as r = 2.96 Å, q = +3.0, and van der Waals well depth of 0.056 kcal/mol. The ligands were prepared for docking by minimizing their energy using B3LYP in combination with the 6-31+G(d,p) basis set for C, H, N, O, Cl, and H, and def2-TZPV basis set for Ru atom, which is implemented in the Gaussian 09 software [37]. The protein-ligand docking was done using the Lamarckian Genetic Algorithm (LGA) method [54]. The grid centers with dimensions 95.32 × −84.16 × 1.80 Å3, 1.78 × 50.98 × 19.67 Å3, and 10.25 × 18.08 × 122.39 Å3 in -x, -y, and -z directions of the ACHE, MM-9, and BSA were used to cover the protein binding sites and accommodate ligands to move freely. The binding affinity of title molecules was investigated and discussed.

### 2.7. HSA-Complexes Interaction Investigated by Electron Paramagnetic Resonance Spectroscopy

#### 2.7.1. Sample Preparation

In order to investigate the nature of the interaction between the two Ru-complexes and human serum albumin (HSA), spin-probing/electron paramagnetic resonance methodology was used additionally. Namely, as HSA can bind up to seven molecules of long-chain fatty acids [55,56], and their doxyl derivatives [57,58,59,60,61,62], which are EPR-active compounds, HSA was first incubated with the fivefold concentration of 16-doxyl-stearic acid (16-DS, Sigma-Aldrich), and subsequently the aliquots of the Ru-complexes solutions were added to the HSA/16-DS mixture. For this experiment, HSA was dissolved in the 100 mM phosphate buffer, pH 7.4, and its concentration was 0.2 mM. The ethanol solution of 16-DS was transferred to the bottom of the test tube, and ethanol was dried in the Eppendorf Concentrator plus vacuum centrifuge. After the ethanol completely evaporated, an appropriate amount of HSA solution was added to the tube containing 16-DS. The concentration of the 16-DS in HSA/16-DS solution was 1 mM, i.e., [HSA]:[16-DS] molar ratio was 1:5. This stock solution was incubated until the full binding of 16-DS to HSA was achieved. The incubation involved occasional gentle vortexing and thermostating at 37 °C in a water bath.

In the next step, Ru(II) complexes were dissolved in DMSO and diluted using 100 mM phosphate buffer, pH 7.4. The concentrations of both Ru(II) complexes stock solutions were 266.67 µM. Small volumes of these solutions were added to HSA/16-DS preparation and diluted with the phosphate buffer. Afterward, the samples were incubated for 30 min before the EPR measurement. Concentrations of HSA and 16-DS in the final samples were 50 µM and 250 µM, respectively, while [HSA]:[Ru-complex] molar ratios were 1:0.5, 1:1, 1:2, and 1:4. The sample with the highest drug concentration (molar ratio 1:4), and hence, the highest concentration of DMSO contained 1.2% *v*/*v* of DMSO. Thus, additional samples containing HSA/16-DS and 1.2% *v*/*v* DMSO were prepared for reference. All of the samples were prepared in duplicate.

#### 2.7.2. EPR Spectra Acquisition and Processing

The samples prepared as previously described were aspirated into the capillary gas-permeable Teflon tubes (Zeus Industries, Inc., Orangeburg, SC, USA) and placed into the resonator cavity of the X-band EPR spectrometer Bruker Elexsys II E500. The EPR spectrometer parameters were as follows: microwave frequency 9.85 GHz, central field 3510 G, sweep width 150 G, microwave power 10 mW, conversion time 117.19 ms, modulation amplitude 2 G, modulation frequency 100 kHz, number of points 1024. The acquired EPR spectra were analyzed using the Bruker Xepr software and simulated by SimLabel [63], a graphical user interface software that is an extension of EasySpin [64], the EPR simulation package (the version of EasySpin used was 6.0.6). All the EPR spectra were deconvoluted into three spectral components corresponding to the strongly bound (SB), weakly bound (WB), and unbound (UB) 16-DS. First, the simulation parameters were optimized by simulating the EPR spectra of samples containing only HSA and 16-DS, using the same initial A- and g-tensor values for all three components found in [65]. The optimal parameters were used as initial parameters to simulate all the other spectra. The simulations were run for each spectrum until the root mean square deviation was less than 0.004.

### 2.8. In Vitro Activity

#### 2.8.1. Reagents and Chemicals

Dulbecco’s Modified Eagle’s Medium (DMEM), penicillin-streptomycin solution, 0.25% trypsin/EDTA, and phosphate-buffered saline were purchased from Gibco. Fetal bovine serum (FBS) was purchased from Capricorn Scientific MTT (3-(4,5-Dimethylthiazol-2-yl)-2,5-Diphenyltetrazolium Bromide) was purchased from Invitrogen, Thermo Fisher Scientific. Thiobarbituric acid and 1,1,3,3-tetramethoxypropane were purchased from Merck. Anti-annexin V-FITC (556419) or Annexin V-APC antibody (550475) were purchased from BD Pharmingen.

#### 2.8.2. Cell Culture and Experimental Designs

The human breast cancer cell line (MDA-MB-231) and human pancreatic cancer cell line (MIA PaCa-2) were obtained from the American Type Culture Collection (ATCC). The cells were cultured in 25 cm^2^ flasks with DMEM supplemented with 100 U/mL penicillin-streptomycin, 10% FBS, at 37 °C with 5% CO_2_ in a humidified incubator.

#### 2.8.3. MTT Test

Cells were harvested when they reached 85–90% confluent. 100 µL of cell suspension (15.000 cells per well) was poured into the wells of the 96-well plate and incubated overnight to allow the cells to attach to the well. The cells were treated with **1** or **2** in concentrations ranging from 0.19 to 200 µM. Untreated cells were used as control. After 24 h, 10 µL MTT (5 mg/mL) was added to each well. Cultures were incubated for 24 h at 37 °C. The reduction process was stopped by adding 100 µL/well of 10% SDS acidified with 1N HCl. Dissolution of the resulting blue color was continued overnight in the incubator at 37 °C. All treatments were done in replicates of three. The absorbance was read at 540 nm on an ELISA reader (RT-6100, Rayto, Shenzhen, China). The ratio of absorbances of treated to untreated living cells was used to measure viability. Cytotoxic activity was recorded as IC_50_, which is the concentration necessary to reduce the absorbance of treated cells by 50% compared to the control [66].

#### 2.8.4. Determination of the Malondialdehyde and Protein Carbonyl Concentrations

MDA-MB-231 and MIA PaCa-2 cells were treated with 50 µM of complex **2** or 75 µM and 100 µM of complex **1**, respectively, for 24 h. Untreated cells were used as control. The supernatants were collected and used for further analyses. Lipid peroxidation was estimated by a spectrophotometric assay based on the absorption maximum of the malondialdehyde (MDA) complex with thiobarbituric acid in an acidic environment and high temperature. 1,1,3,3-Tetramethoxypropane was used as standard. Absorbance was read on the ELISA reader using a 540 nm filter. The concentration was determined using a standard curve [67]. The determination of protein carbonyl (PC) concentration was based on the method of Levin and associates [68]. Absorbance was read on the ELISA reader using a 375 nm filter.

#### 2.8.5. Detection of Matrix Metalloproteinase 2 and 9 Activity

MDA-MB-231 and MIA PaCa-2 cells were treated with 50 µM of complex **2** or 75 µM and 100 µM of complex **1**, respectively. The treatments lasted 24 and 48 h. Untreated cells were used as control. The supernatants were collected for further analysis. Electrophoresis in a polyacrylamide gel with an add-on of 0.1% SDS and 0.2% gelatin was used to measure metalloproteinase 2 and 9 activity (MMP2 and MMP9).

The gels were imaged with a ChemiDoc Imaging System (Bio-Rad Laboratories, Hercules, CA, USA) and estimated by densitometric scanning using the Image Lab (Bio-Rad Laboratories, Inc. Version 6.0.0.25) software tool.

#### 2.8.6. Annexin V/PI Apoptotic Assay

MDA-MB-231cells were treated with 50 µM of complex **2** and 75 µM of complex **1**, while MIA PaCa-2 were treated with 50 µM of complex **2** and 100 µM of complex **1** for 24 h. After treatment, the cells were washed once in PBS, once in Annexin Binding Buffer (14 mM NaCl, 0.4 mM KCl, 75 µM MgCl_2_, 1 mM HEPES), and resuspended in 100 μL of Annexin Binding Buffer. RNA was removed by adding 10 µg/µL RNAse A and incubating the cells for 1 h at 37 °C. Then, 5 μL of anti-Annexin V-FITC or Annexin V-APC antibody was added, and cells were incubated for 30 min at 4 °C in the dark. Unbound antibody was washed with PBS. Cells were resuspended in 500 μL, stained with 25 µg/µL PI (ThermoFisher Scientific, P1304MP, Waltham, MA, USA), and read at the BD FACSCallibur flow cytometer. Data was analyzed using FloJo v10.8.1 software.

#### 2.8.7. Statistical Analysis

The One-way ANOVA and LSD posttest were applied using SPSS software for Windows (SPSS 16.0 for Windows Evaluation Version software, SPSS Inc., USA). The results are expressed as the mean ± SEM, and differences at *p* < 0.05 are accepted as the significance level.

## 3. Results and Discussion

### 3.1. Synthesis and Spectral Characterization of Complexes ***1*** and ***2***

**Scheme 1:**
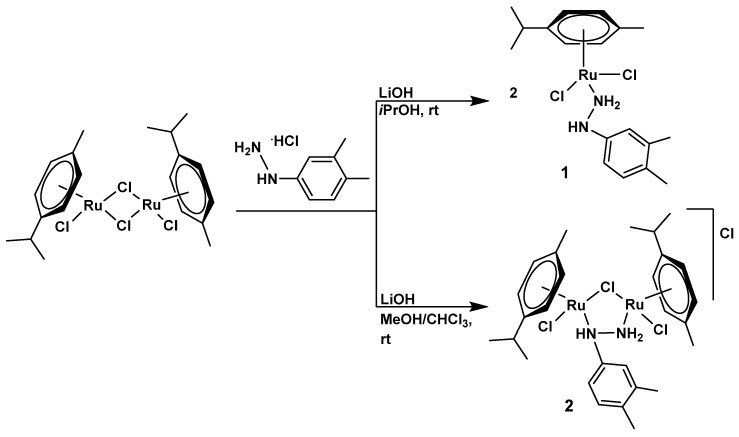
Synthesis of Ru(II) complexes **1** and **2**.

The synthesis of both the mononuclear complex [RuCl_2_(η^6^-*p*-cymene)(3,4-dmph-κ*N*)] (**1**) and the binuclear complex [{RuCl(η^6^-*p*-cymene)}_2_(μ-Cl)(μ-3,4-dmph-κ^2^*N*,*N*′)]Cl (**2**; 3,4-dmph = 3,4-dimethylphenylhydrazine) was achieved successfully in 2-propanol or methanol/chloroform (4:1), depending on the reaction conditions (Scheme 1). The binding mode of the 3,4-dimethylphenylhydrazine ligand observed in these complexes is noteworthy and aligns with previously reported structures [69], though more sterically hindered aromatic hydrazines like this one have not been extensively explored in “piano-stool” Ru(II) complexes until now.

This research highlights the versatile coordination behavior of hydrazine ligands, which is highly influenced by reaction conditions, including the choice of solvent and reagent ratios. This adaptability allows for precise control over the transition between bridging and monodentate coordination modes, facilitating the customization of complex properties. Notably, incorporating bridging hydrazine, stabilized by chlorido ligands and hydrogen bonding, results in bio-reactive complexes. These characteristics make the complexes promising candidates for biological applications, with the potential for further functionalization by adding ligands to one or both Ru(II) centers.

In the ¹H NMR spectrum of the mononuclear complex **1** (Figure S1), the aromatic protons of the 3,4-dmph are observed as broad resonances in the range of 7.02–6.71 ppm, which can be attributed to weak long-range couplings. The NH and NH_2_ groups of the hydrazine moiety produce distinct chemical shifts at 5.29 ppm and 2.19 ppm, respectively, with slight broadening. The doublets at 5.54 ppm and 5.34 ppm are assigned to the aromatic protons of the *p*-cymene ring, while the methyl groups on the *p*-cymene are represented by resonances at 1.33 ppm and 2.18 ppm. Additionally, the heptet at 3.04 ppm corresponds to the central proton of the isopropyl group in the *p*-cymene moiety. The chemical shifts of methyl hydrogen atoms from the 3,4-dmph resonate at 2.21 and 2.19 ppm.

Two sets of resonances are observed for the binuclear complex **2** (Figure S2) due to the building of diastereosimers upon coordination of 3,4-dmph to two Ru(II) centers in the ^1^H NMR spectrum of **2**. The NH and NH_2_ protons of the hydrazine moiety produce two sets of signals at 5.38/5.37 ppm and 5.27/5.25 ppm. The aromatic protons of 3,4-dmph and the *p*-cymene are found between 8.57–7.67 and 5.69–5.54. Chemical shifts for the methyl hydrogen atoms on the *p*-cymene and 3,4-dmph appear at 1.38–1.04 and 2.16–1.88 ppm, while the central proton of the isopropyl chain gives rise to a heptet at 2.91 and 2.67 ppm.

In the ¹³C NMR spectrum of **1** (Figure S1), the resonances for the aromatic carbon atoms of the *p*-cymene moiety are observed between 102.6 and 60.6 ppm, while the carbon atoms of the hydrazine aromatic ring resonate in the range of 146.6–113.1 ppm. The methyl groups of the *p*-cymene structure give rise to chemical shifts at 18.7 ppm, while the isopropyl group carbon atoms appear at 22.4 ppm (CH). Methyl groups from 3,4-dmph resonated at 19.9 and 19.0 ppm.

For the binuclear complex **2** (Figure S2), the aromatic carbons of the 3,4-dmph ligand appear between 145.7–119.2 ppm, while the aromatic carbons of the *p*-cymene structure resonate in the region of 105.5 and 76.6 ppm. The methyl group carbon atoms of the *p*-cymene are located at 16.5 and 16.3 ppm, while the isopropyl group carbons produce signals at 30.0 and 29.8 ppm (CH). Resonances at 20.1 and 19.1 ppm are assigned to methyl carbon atoms from the 3,4-dmph ligand.

The stability of complexes in 1 × 10^−4^ M solutions in PBS was followed by UV-VIS spectroscopy. The spectra are shown in Figure S3 for the measurements between 0 and 24 h. After dissolution, both complexes have two very intense absorption bands between 200–350 nm, due to ligand-centered *π*→*π*^∗^ and n→*π*^∗^ transitions. At around 400 nm additional bands were detected assigned to metal→ligand and ligand→metal charge transfers, as well as *d*-*d* transitions [70,71]. The time-dependent spectra indicated ligand-substitution reactions at the Ru(II) center. The intensity of this band in the case of complex **2** is much higher due to the presence of two central metal ions. There is a gradual decrease in the absorbance at 400 nm and an increase in the band at 311 nm. Similar (η^6^-arene)Ru(II) complexes are known to undergo a monoexponential decrease in absorbance due to the hydrolysis, as observed herein for complex **1**, thus loss of one chloride ligand and substitution by water molecule [72]. Binuclear (η^6^-arene)Ru(II) complexes may undergo dissociation in solution, leading to the formation of mononuclear species along with the coordination of solvent molecules [73]. In the time-evolution spectra of complex **2**, there is a gradual increase in absorbance, likely due to hydrolytic cleavage accompanied by the hydrolysis of the binuclear complex.

### 3.2. Structural Characterization of Complex ***2b***

**Figure 1:**
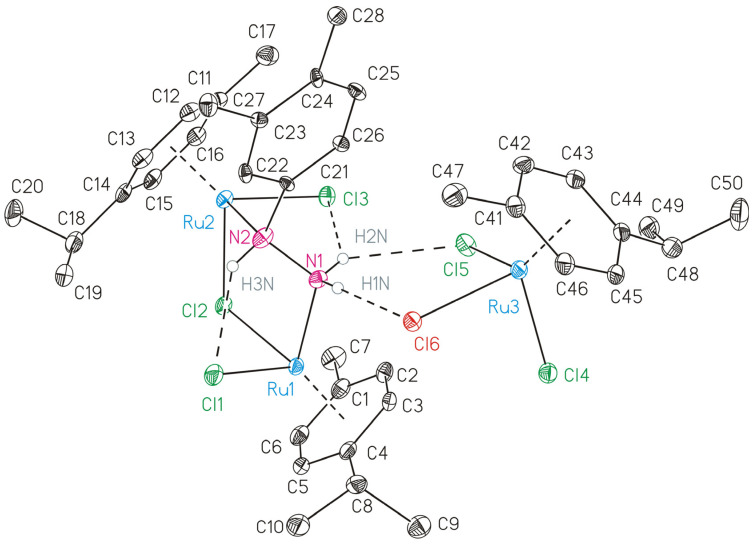
ORTEP (30% probability ellipsoids) of the hydrogen bridged ion pair of [{RuCl(η^6^-*p*-cymene)}_2_(μ-Cl)(μ-3,4-dmph-κ^2^*N*,*N*′)]^+^ (left) and of [RuCl_3_(*p*-cymene)]^−^ (right). All carbon-bonded hydrogens and disordered atomic positions are omitted for clarity. Dashed lines represent aryl Ruthenium interactions as well as hydrogen bonds.

The crystal structure of **2b** was obtained unexpectedly during attempts to crystallize the cationic binuclear Ru(II) complex **2** from a solution in chloroform. The compound **2b** crystallizes in the triclinic crystal system with the space group **P-1**. The crystal structure of **2b** comprises a cationic binuclear Ru(II) complex, [{RuCl(η^6^-*p*-cymene)}_2_(μ-Cl)(μ-3,4-dmph-κ^2^*N*,*N*′)]^+^ ((**2**–Cl)**^+^**)**,** paired with a mononuclear anionic complex [RuCl_3_(*p*-cymene)]^−^ which interact through hydrogen bridges (Figure 1, Tables S1–S3).

The cationic binuclear complex features two ruthenium centers bridged by a 3,4-dmph hydrazine ligand. The nitrogen atoms of the 3,4-dmph ligand coordinate asymmetrically to the ruthenium atoms, with Ru–N bond lengths of 2.136(5) Å and 2.183(4) Å and an N–N bond length of 1.466(7) Å, indicative of partial double-bond character. Each Ru(II) center adopts a “piano-stool” geometry coordinated to an aromatic ring, a chlorido ligand, and the bridging hydrazine. The Ru–Cl distances for the chlorido ligands are in the range of 2.4007(15)–2.4481(14) Å. The anionic mononuclear complex, [RuCl_3_(*p*-cymene)]^−^, exhibits a typical “piano-stool” configuration, with the Ru(II) center bound to the p-cymene ligand and three terminal chlorido ligands. The Ru–Cl bond lengths fall within the expected range for such complexes, ensuring stability through strong Ru–Cl interactions [69]. The bridging 3,4-dmph ligand plays a central role in maintaining the structure of the cationic binuclear complex, while the mononuclear anion [RuCl_3_(*p*-cymene)]^−^ contributes to the electrostatic balance and overall stability of the crystal.

### 3.3. Quantum-Chemical Analysis of Structure and Stability of Complexes ***1*** and (***2***–Cl)**^+^**

**Figure 2:**
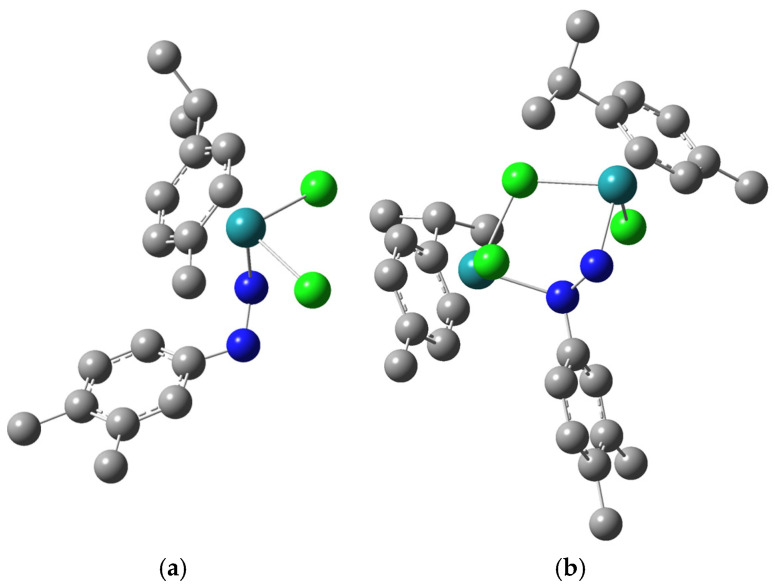
Optimized structures of complexes **1** (**a**) and (**2**–Cl)**^+^** (**b**) at B3LYP/6-311++G(d,p)(H,C,N,Cl)/def2-TZVP level of theory (The hydrogen atoms are omitted for clarity, carbon-gray, nitrogen-blue, chlorine-gray, ruthenium-teal).

The structures of complex **1** and complex cation (**2**–Cl)**^+^** were optimized at B3LYP/6-311++G(d,p)(H,C,N,Cl)/def2-TZVP(Ru) level of theory. The same functional was previously applied for the optimization and spectral characterization of mono- and binuclear Ru(II) complexes with similar ligands [69,74]. The structures presented in the mentioned references served as a starting guess for the structural characteristics of the compounds from this contribution. The optimized structures of complexes **1** and (**2**–Cl)**^+^** are presented in Figure 2. As the crystallographic structure of (**2**–Cl)**^+^** was available, as previously explained, the experimental and optimized bond lengths were compared by calculating the correlation coefficient (R) and the mean absolute error (MAE). The experimental and theoretical bond lengths and angles of (**2**–Cl)**^+^** are given in Tables S1 and S2.

The structure of examined complexes consists of *p*-cymene and dimethylphenylhydrazine moieties. These groups contain extended delocalization, and significant differences between experimental and theoretical values of bond lengths and angles are not expected, as previously proven in [69,74]. In complex **1**, the Ru−Cl bond lengths are 2.44 and 2.43 Å, which is in the range of values experimentally obtained for similar complexes [74]. It should be noted that the equilibration of these bond lengths is a consequence of the optimization process and pseudo-octahedral geometry around the central metal ion. The Ru−N bond is 2.18 Å, again very similar to the crystallographic value for complexes with nitrophenylhydrazine and chlorophenylhydrzine ligands [74]. The angle Cl−Ru−Cl is 88.92°, while the angles enclosed by nitrogen, ruthenium, and chlorine atoms are 78.92 and 80.60°. These values are consistent with previously mentioned complexes, proving that the additional substituents on phenylhydrazine do not affect the overall geometry of the complex, which is a consequence of the delocalization within this group. When complex **2** is concerned, the Ru−Cl bond lengths are between 2.29 and 2.48 Å. The bonds between the chlorido bridge and Ru(II) ions are slightly longer than those with other chloride atoms. The binuclear Ru(II) complex with napthylhydrazine ligand was characterized by a similar range of values. The bonds between the nitrogen atom and Ru(II) are also similar (2.18 and 2.21 Å), although the position of the nitrogen atom differs in the ligand structure. This result leads to the conclusion that local symmetry within the structure is preserved. The same is appropriate when discussing angles between chlorine, ruthenium, and nitrogen atoms. Their values are between 82 and 85°. The applicability of the selected theory level was proven upon comparing the experimental and theoretical bond lengths and angles of (2–Cl)+, with the correlation coefficients being higher than 0.999 and MAE values equal to 0.025 Å and 1.50°.

The ^13^C NMR chemical shifts were calculated for the optimized structures. The experimental, unscaled, and scaled theoretical values are shown in Tables S4 and S5. High correlation coefficients (0.999 (**1**) and 0.992 (**2**)) and low MAE values (1.55 (**1**) and 2.88 (**2**) ppm) verify that the optimized structures resemble the experimental ones. These results led to further use of these optimized structures to investigate stabilization interactions and protein binding properties.

The QTAIM analysis was applied to examine the stabilization interactions between the central metal ions and surrounding donor atoms, as suggested in references [75,76,77]. These interactions are depicted in Figure S4. The presence of *p*-cymene moiety is significant for structure stability, as concluded by theoretical and spectroscopic methods in [69,74]. Four interactions exist between carbon atoms of this moiety and Ru(II) in the structure of complex **1**. These interactions are characterized by electron density of around 0.080 a.u. and Laplacian of 0.2400 a.u, which classifies this interaction as open-shell. This is consistent with other half-sandwich Ru(II) complexes [69,78]. The values of the ratio between kinetic and potential electron density of these interactions are 0.8, proving their partial covalent character. The interatomic bond energies are between −127.7 and −133.0 kJ mol^−1^, among the highest values of those presented in Table S6. The interatomic bond energies were calculated as the half value of the potential electron density value, according to the reference [79]. Interactions between chlorine and Ru(II) have lower electron densities (0.066 a.u.) and Laplacians (0.185 a.u.), with -G(r)/V(r) that are still lower than 1. The Ru−Cl bond energies are −91.8 and −136.8 kJ mol^−1^. Similar values were calculated for the interaction with a nitrogen atom that has partial covalent character (-G(r)/V(r) = 0.8) and bond energy of −93.8 kJ mol^−1^. The structure of complex **1** is additionally stabilized by a strong hydrogen bond denoted as Cl∙∙∙H−N, with a bond energy of −12.8 kJ mol^−1^. The presence of hydrogen atoms attached to aromatic rings leads to the formation of weak hydrogen bonds of the type Cl∙∙∙H−C with much lower bond energies of −5.2 and −3.1 kJ mol^−1^. The interactions between partially positively charged hydrogen atoms and π-electron cloud are reflected in the formation of C∙∙∙H−C, with bond energies of −3.2 and −1.2 kJ mol^−1^. The mentioned weak interactions have -G(r)/V(r) values higher than 1.

The complex cation (**2**–Cl)**^+^** structure has three bond critical points between carbon atoms of *p*-cymene and Ru(II) for each of the central metal ions. These interactions have the same energies as previously discussed. A significant difference is in reduced bond energies of Ru−Cl interactions of around −90 kJ mol^−1^, while the energies of Ru−N increase to 122.7 and −139.1 kJ mol^−1^ (Table S6). This proves the bridging ligand’s importance for the binuclear complex’s stability. All of these interactions have partial covalent character. Due to the larger number of other moieties in the binuclear complex, the number of weaker interactions is increased, as shown in Table S3. These interactions again include weak hydrogen bonds (Cl∙∙∙H−C) with energies between −24.9 and −3.4 kJ mol^−1^. These higher energies result from the proximity of chlorine atoms and partially positive hydrogen atoms of aromatic groups. The weakest interaction is denoted as H∙∙∙C−H with an energy of −1.4 kJ mol^−1^.

### 3.4. Binding Studies of Complexes ***1*** and ***2*** with HSA

HSA is a major transport protein in the human body. It consists of three domains (I, II, and III) divided into three subdomains (A and B). These subdomains contain cavities that are responsible for the transportation of fatty acids and other important compounds. The fluorescence emission of HSA, which has been exploited in this work, originates from the amino acid tryptophan in position 214 [80]. This amino acid is positioned between domains IIA and IIB in the so-called FA8 binding site. This region includes two helices that form a sharp angle, limiting the size of the compounds directly interacting with Trp214.

**Figure 3:**
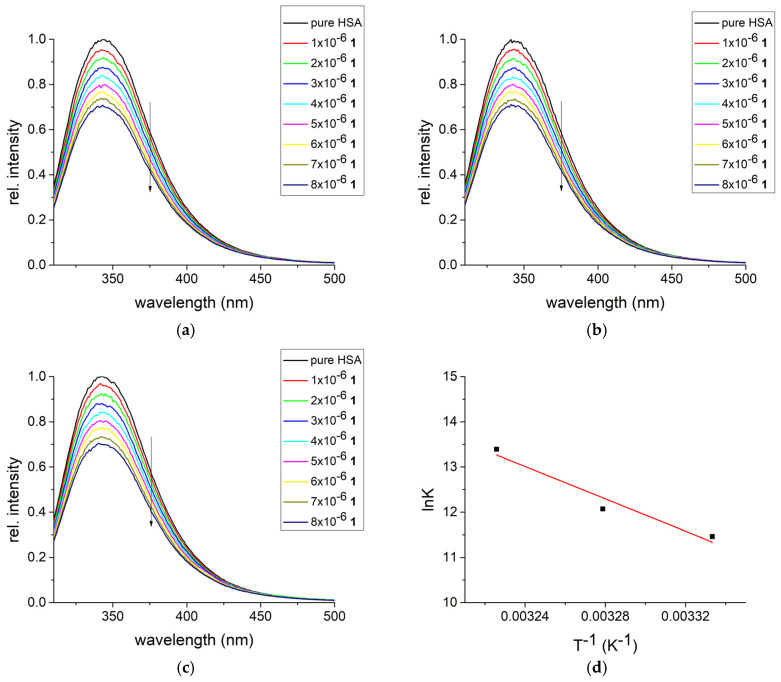
The fluorescence emission spectra of HSA for the titration with various concentrations of complex **1** at (**a**) 27°, (**b**) 32°, and (**c**) 37 °C, and (**d**) the van ’t Hoff plot for the binding process.

The interactions between transport protein and compounds **1** and **2** were followed by spectrofluorometric titration, as shown in references [81,82]. The emission spectra of HSA with and without added complexes are shown in Figure 3 and Figure S5. The addition of complexes leads to a decrease in the fluorescence spectra intensity in a concentration-dependent manner, similar to measurements involving other ruthenium complexes in [83,84]. The binding constant, number of binding positions, and correlation coefficients for the dependency according to Equation (1) are presented in Table 1. The linearity of the graph is proven by the high correlation coefficients, between 0.995 and 0.999. The binding constants are in the range between 9.50 × 10^−4^ (27 °C) and 6.53 × 10^−5^ M^−1^ (37 °C) for complex **1** and between 2.95 × 10^−4^ (27 °C) and 8.19 × 10^−4^ M^−1^ (37 °C) for complex **2**. It should be noted that the range is much narrower for complex **2**, which shows that the binding process is less dependent on temperature.

When measurements at three temperatures are compared, the thermodynamic parameters of binding can be calculated. The change in enthalpy and entropy of binding in the case of complex **1** is 149.8 kJ mol^−1^ and 590 J mol^−1^ K^−1^. The positive value of the enthalpy change is characteristic of the endothermic processes and includes fewer favorable interactions, such as weaker electrostatic and hydrogen bonds. Also, positive changes in enthalpy can be observed in hydrophobic interactions or conformational changes in protein structure that require additional energy. On the other side, positive change in entropy is associated with the binding processes in which water molecules are released from the protein structures, leading to increased disorder in the system. Therefore, the binding of complex **1** to HSA is driven by entropy. These positive values were also found for the interactions between similar Ru(II) complex ([RuCl(η^6^-*p*-cymene)(3-DNPH)] (chlorido(η^6^-*p*-cymene)(3-nitrophenylhydrazine-k^2^*N*,*N*’)ruthenium(II)) and Bovine Serum Albumin (BSA) in reference [74]. The change in Gibbs binding energy of the process ranges from −28.3 to −34.2 kJ mol^−1^, marking the binding process as spontaneous in the examined temperature range. The thermodynamic parameters are lower for the second complex, namely ΔH_b_ = 78.9 kJ mol^−1^ and ΔS_b_ = 348 J mol^−1^ K^−1^. The overall spontaneity of the binding process is lowered, as well. The change in Gibbs free energy of binding is between −25.6 and −29.1 kJ mol^−1^. The interactions between another binuclear Ru(II) complex with 1-naphthtylhydrazine ligand and BSA were also spontaneous with ΔG_b_ values between −29 and −34 kJ mol^−1^ [69]. It can be assumed that the size of complex (**2**–Cl)^+^ cation limits the number of favorable interactions within the active pocket of HSA. A detailed examination of the interactions at the molecular level is presented in the last section of the article.

### 3.5. HSA-Complexes Interaction Assessed by EPR Spectroscopy/Spin-Probing Methodology

**Figure 4:**
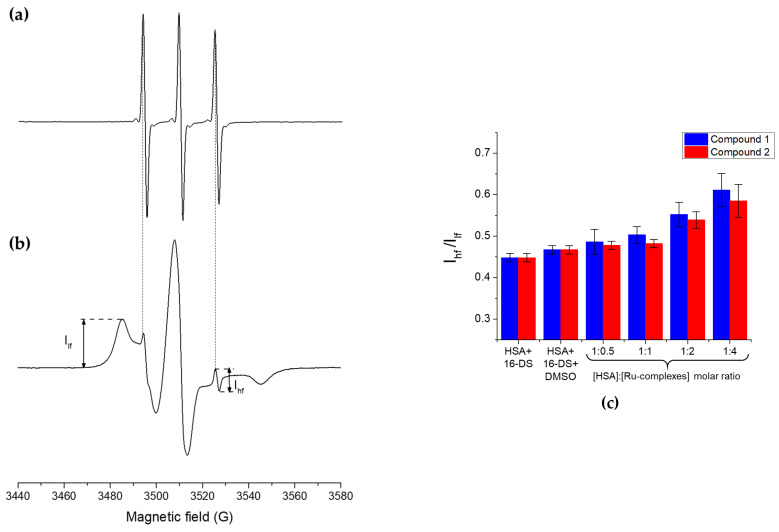
EPR spectra of 250 µM 16-DS: (**a**) in the phosphate buffer (100 mM, pH 7.4), and (**b**) incubated with HSA at [HSA]:[16-DS] molar ratio of 1:5. The spectra are not presented on the same intensity scale. The markers show the amplitudes of low-field (I_lf_) and high-field peak (I_hf_) that correspond to the bound and unbound 16-DS, respectively. (**c**) The amplitudes ratio of the high-field (I_hf_) and low-field (I_lf_) peaks calculated for the different molar ratios of [HSA]:[Ru-complexes]. All samples were prepared in the phosphate buffer (100 mM, pH 7.4) and contained 50 µM HSA, 250 µM 16-DS. The reference sample HSA+16-DS+DMSO contained 1.2% *v*/*v* DMSO, the same amount as the sample at the [HSA]:[Ru-complex] molar ratio 1:4.

HSA contains seven well-described binding pockets for the long-chain fatty acids, designated FA1-FA7 [55,56], among which FA1-FA5 are sites with higher binding affinity due to the formation of salt bridges and hydrogen bonds with the carboxylic group of fatty acids, unlike sites FA6-FA7, that are considered the sites of the weakest binding [55,56,85]. FA4 and FA5 are the highest affinity binding sites, followed by FA2, a site of medium affinity, and FA1 and FA3, the lower affinity binding sites. Furthermore, the doxyl-derivatives of stearic acid (DS) have also been shown to bind to HSA even at very high [HSA]:[DS] molar ratios (above 1:5) [57,59,60,61]. Thus, in the EPR/spin-probing experiments presented in this work, the HSA was incubated with 16-doxyl-stearic acid (16-DS) at a molar ratio of 1:5, to investigate whether the Ru-complexes are able to displace the 16-DS molecules already bound to HSA, which would imply an interaction between HSA and Ru-complexes. The advantage of using EPR methodology for the competitive binding experiments lies in the fact that EPR spectra of the 16-DS, when mixed with albumin, simultaneously provide information on both bound and unbound 16-DS molecules. The typical spectrum of the sample containing only HSA and 16-DS at [HSA]:[16-DS] molar ratio of 1:5 is depicted in Figure 4b. The presented spectrum is the sum of the EPR spectra corresponding to the 16-DS freely tumbling in an aqueous solution (unbound component, UB) given as a narrow sharp triplet (Figure 4a), and the much broader component arising from the 16-DS molecules bound to the various binding sites in HSA. The marked amplitudes of the high-field (I_hf_) and low-field (I_lf_) peaks depend on the amount of unbound and bound 16-DS, respectively. Moreover, the spectral component of the 16-DS bound to HSA is comprised of two components—strongly bound (SB) and weakly bound (WB) 16-DS [57,58,86,87]. All three components can be extracted from the experimental spectra by performing EPR spectral simulations using the software developed specifically for this purpose [63,64].

As evidenced from the spectra presented in Figure S6, upon adding both Ru-complexes, it can be observed that the amplitude of the high-field peak, I_hf_, increases. Although the Figure S6 shows the spectra of HSA/16-DS/Ru-complexes samples at [HSA]:[Ru-complex] ratio of 1:4, the effect is observable even at the lower [HSA]:[Ru-complexes] molar ratios. This would indicate that to a certain, rather small extent, 16-DS molecules may be displaced in the presence of both Ru-complexes, implying that they bind to HSA, although probably with low affinity. Among the two complexes, the effect seems to be more pronounced in complex **1**, inferring that this complex binds to HSA with somewhat greater affinity than complex **2**. The amount of the displaced 16-DS can be estimated by performing the deconvolution of the spectra using the EPR spectral simulations since one of the many output parameters is a contribution of each of the three aforementioned spectral components. Figure S7 shows the spectral components obtained for the sample containing only HSA and 16-DS, while Table 2 summarizes the contributions of the unbound 16-DS to the total spectra. The contribution of the unbound 16-DS exhibited only a minor change (approx. 0.2%) at the highest [HSA]:[Ru-complexes] molar ratio (1:4), compared to the reference samples (HSA/16-DS and HSA/16-DS/1.2% *v*/*v* DMSO).not shown.

Although EPR spectral simulations provide relatively accurate quantitative information about the amount of unbound/weakly-/strongly bound spin-probe, the alterations in the EPR spectra caused by mixing the HSA/16-DS with Ru-complexes are very subtle and may become more obvious if the I_hf_/I_lf_ is used, instead (see Figure 4c). It should be highlighted that this parameter can only be used to observe the alterations in the spectral features qualitatively, as the amplitudes of these peaks are not a quantitative measure of the bound and unbound spin-probe since these two components exhibit substantially different shapes and spectral widths. Thus, their amplitudes cannot be compared, as previously discussed [57,60]. From the measured parameters, it can be seen that the amplitude of the low-field peak decreases with the enhancement of the concentrations of both Ru-complexes while the I_hf_/I_lf_ increases (Figure 4c). Complex **1** exerts a more pronounced increase of the I_hf_/I_lf_ parameter than complex **2**. This trend aligns with the results obtained by simulations and leads to the conclusion that both complexes can displace 16-DS from the preformed HSA/16-DS protein/ligand complex, indicating that they both bind to HSA. However, the extent of 16-DS displacement is minor, suggesting that both complexes bind to HSA with low affinity, or at least lower than the weakly bound molecules of 16-DS.

### 3.6. In Vitro Activity

#### 3.6.1. MTT Test

**Figure 5:**
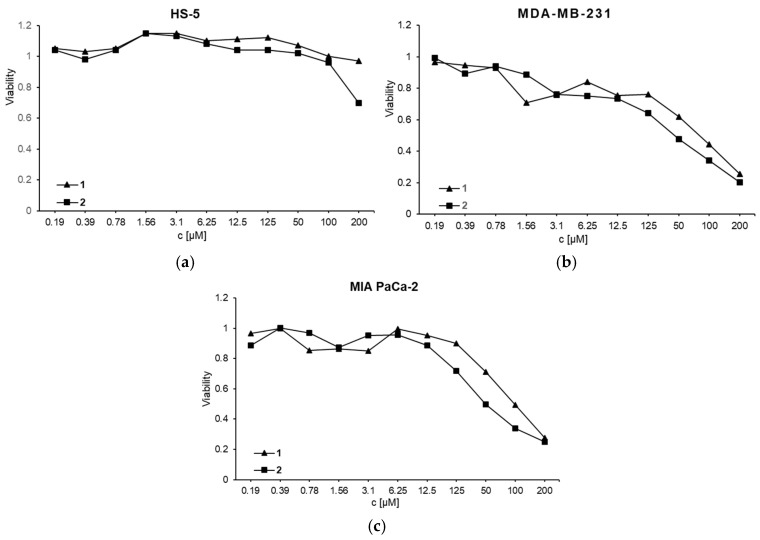
Viability of (**a**) HS-5, (**b**) MDA-MB-231, and (**c**) MIA PaCa-2 cancer cells under the effect of **1** and **2** for 24 h of treatment (MTT assay).

The cytotoxicity of complexes **1** and **2** were tested against MDA-MB-231 and MIA PaCa-2 cancer cell lines. Cell cultures were exposed to different concentrations of **1** and **2** ranging from 0.19 to 200 µmol (Figure 5). Untreated cells were used as control. The MDA-MB-231 and MIA PaCa-2 cell viability was decreased in a dose-dependent manner where the IC_50_ concentration for **1** was 75 µM and 100 µM, respectively, for **2** was 50 µM on both investigated cell lines.

The results obtained on both cancer cell lines revealed that complex **2** has a lower IC_50_ concentration than complex **1**. At the same time, it is evident that **1** has a more significant effect on the viability of breast adenocarcinoma cells compared to pancreatic cancer cells. To check the cytotoxicity of complexes on healthy cells, fibroblast-like HS-5 cells were treated with the same concentrations of **1** and **2** for 24 h. It was found that the concentrations of **1** and **2** used in experiments with MDA-MB-231 and MIA PaCa-2 did not affect the viability of HS-5 cells (Figure 5). This selectivity suggests that certain cancer types may exhibit differential sensitivity to treatment and that complexes **1** and **2** may have a favorable therapeutic profile, effectively targeting cancer cells while sparing healthy cells. This sparing effect is significant, as it implies that both **1** and **2** may effectively target cancer cells while minimizing potential side effects associated with damage to normal tissues, supporting their potential therapeutic profiles.

#### 3.6.2. Malondialdehyde (MDA) and Protein Carbonyl (PC) Concentrations

**Figure 6:**
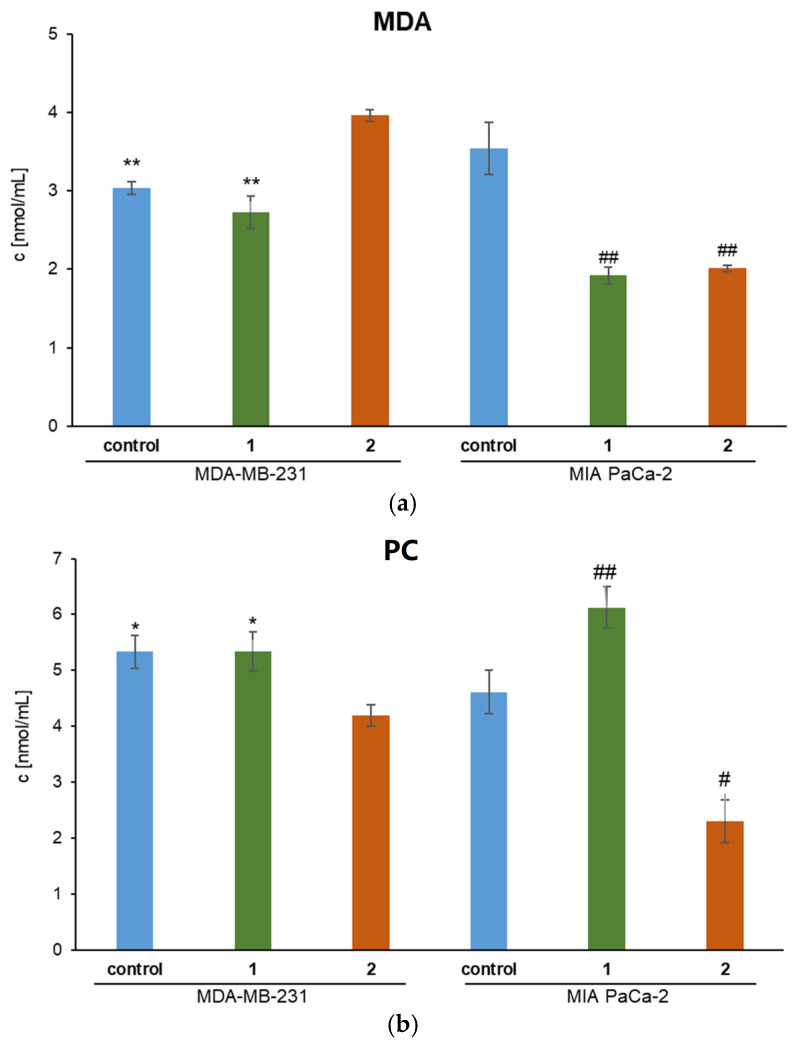
The concentration of (**a**) MDA and (**b**) PC in the medium of treated MDA-MB-231 and MIA PaCa-2 cells. * *p* < 0.05 (comparison to **2**); ** *p* < 0.01 (comparison to **2**); ^#^ *p* < 0.01 (comparison to control); ^##^ *p* < 0.01 (comparison to control).

The concentration of MDA and PC in the medium indicates the prooxidative or antioxidant effect of Ru(II) complexes on cancer cells (Figure 6). Complexes **1** and **2** have a pronounced antioxidant effect in MIA PaCa-2 pancreatic cancer cell culture. MDA concentration is significantly reduced after 24 h treatment with Ru(II) complexes (*p* < 0.01). The concentration of PC in the medium decreased (*p* < 0.05) after treatment with **2**, while **1** increased (*p* < 0.01) the concentration of PC compared to the control. Complex **1** reduces MDA concentration but not PC in MDA-MB-231 breast adenocarcinoma cell cultures. Treatment with **2** has opposite effects on the amount of oxidation products in the medium. Thus, the concentration of MDA is increased (*p* < 0.01), and conversely, the concentration of PC has been decreased (*p* < 0.05) compared to untreated cancer cells.

The results suggest that **1** and **2** may offer protective antioxidant benefits in specific cancer contexts. The findings indicate that **1** and **2** have distinct effects on oxidative stress markers in pancreatic and breast adenocarcinoma cell cultures, suggesting different mechanisms of action. The differential effects on PC levels add complexity to interpreting these results, reflecting the nuanced mechanisms these complexes may operate.

#### 3.6.3. Matrix Metalloproteinase (MMP) 2 and 9 Activity

**Figure 7:**
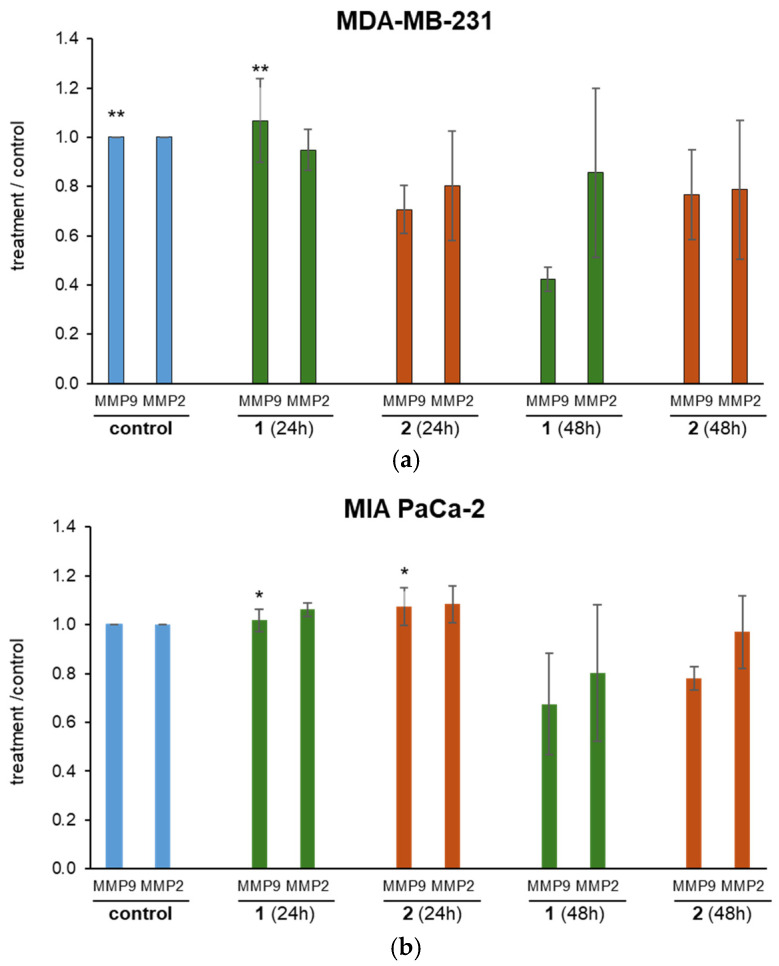
Matrix metalloproteinase 2 and 9 activity in (**a**) MDA-MB-231 and (**b**) MIA PaCa-2 cancer cells under the effect of the complexes **1** and **2**. * *p* < 0.05 (comparison to **1**); ** *p* < 0.01 (comparison to **1**).

Treatment with complex **2** did not affect MMP 9 activity. In contrast, after 48 h, complex **1** causes significantly lower MMP9 activity in breast (*p* < 0.01) and pancreatic (*p* < 0.05) cancer cell cultures compared to control and 24 h treatment (Figure 7). This indicates that the effect of **1** is time-dependent. No changes are detected in MMP2 activity in cell cultures after 24 and 48 h treatments. The observed time-dependent effect suggests that prolonged exposure enhances its inhibitory action, reinforcing the importance of treatment duration in maximizing therapeutic outcomes.

#### 3.6.4. Examination of the Cell Cycle

**Figure 8:**
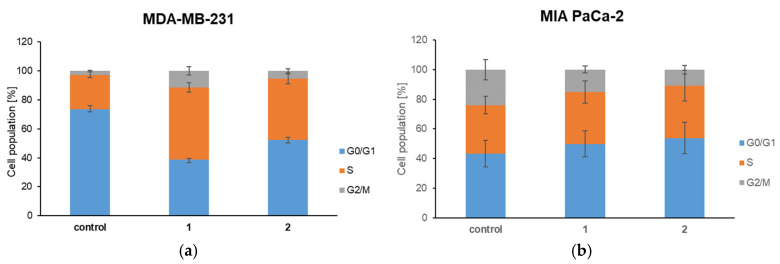
The cell cycle of (**a**) MDA-MB-231 and (**b**) MIA PaCa-2 cells under the effect of complexes **1** and **2**.

The influence of new complexes on the cell cycle is also investigated (Figure 8). The cells were treated for 24 h. Significant changes occurred only in breast adenocarcinoma cells, while the treatment did not affect the pancreatic cancer cells. In the presence of **1** and **2**, the number of cells in the G0/G1 phase significantly decreased while the number of cells in the S phase increased (*p* < 0.01). These results indicate increased proliferation of breast adenocarcinoma cancer cells after treatment.

This proliferative response indicated by the statistical data (*p* < 0.01) raises important questions regarding the underlying mechanisms by which these complexes exert their effects on cell cycle dynamics. In contrast, the lack of response observed in pancreatic cancer cells suggests a selective action of these complexes that may be influenced by the unique cellular and molecular characteristics of different cancer types.

### 3.7. Molecular Docking Studies of Interactions Between Complexes **1** or (**2**–Cl)**^+^** and Proteins

Molecular docking simulations estimated the molecular interactions between amino acids in active binding sites of ACHE, MMP-9, and HSA receptors and examined compounds. The pockets and binding sites of the target receptor were determined. The AGFR software was applied to configure and compute affinity maps for a receptor molecule for further use in AutoDock4.2. The position and orientation of the ligand inside the receptor and the interactions with amino acids bound to the ligand were analyzed and visualized with Discovery Studio 4.0 and AutoDockTools.

The calculated values of free energies of binding and inhibition constants of complexes **1** and (**2**–Cl)**^+^** with investigated receptors were presented in Table 3. The lower value of Ki and more negative value of ΔG_bind_ indicate better inhibition. The inhibitory activities of the selected Ru complexes toward three receptors were ranked based on their lowest binding energy involved in complex formation at the active sites. The binding energies of the title compounds were found to be in the range from −27.1 to −41.5 kJ mol^−1^ (Table 3).

**Figure 9:**
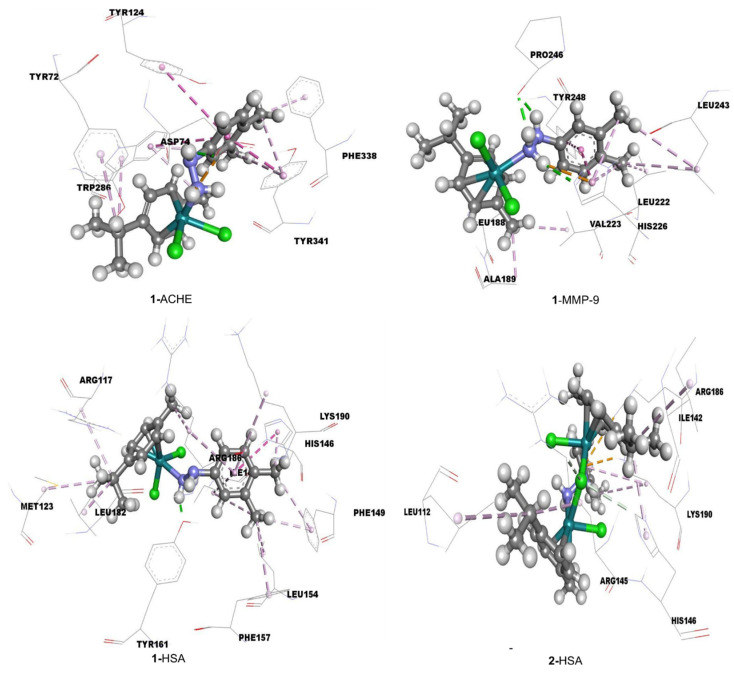
The hydrogen bond (green dotted lines) and hydrophobic (rose pink dotted lines) docking interactions of the most stable conformations of **1** with ACHE, MMP-9, and HSA.

The results presented in Table 3 indicate that the Ru complexes exhibit the most pronounced activity against ACHE and MMP-9 compared to HSA. Notably, complex **1** demonstrates significant binding activity towards MMP-9 and ACHE, whereas the activity of **2** towards other receptors is comparatively weaker. These results indicate that **1** can potentially inhibit ACHE and MMP-9. Figure 9 illustrates the interactions of **1** with ACHE and MMP-9.

The docking analyses revealed that several non-covalent interactions existed between investigated molecules and target receptors. As shown in Figure 9, the most significant interactions include hydrogen bonds, alkyl-π, and π-π interactions. The amino acids that form hydrogen bonds with the ligands play a predominant role in the active sites of these receptors. These amino acids establish strong hydrogen bonds, whereas other amino acids create weaker alkyl-π and π-π interactions with the benzene ring and alkyl moiety of the investigated ligands. The obtained results for the binding energy of complexes **1** and **2** to HSA are in good accordance with the experimental biological data [51].

## 4. Conclusions

In conclusion, this study systematically demonstrates the promising potential of both obtained mononuclear and binuclear Ru(II) complexes as anticancer agents, supported by comprehensive structural and biological evaluations. The synthesis and characterization of these complexes using NMR spectroscopy and FTIR spectroscopy provided valuable insights into their structural integrity and electronic properties, further complemented by DFT calculations that enhanced our understanding of their reactivity.

The bond lengths and angles of the optimized structure of (**2**–Cl)**^+^** were compared to the crystallographic data, and high correlation coefficient and low MAE values proved that the selected level of theory was applicable to the examined structures. The intramolecular interactions showed differences between mono and binuclear complexes. The changes in Gibbs binding energy of the binding process between both complexes and HSA were negative in the investigated range, proving the spontaneity of the process.

Spin-probing results indicated that both complexes can displace 16-DS from the preformed HSA/16-DS protein/ligand complex, although the binding to HSA is characterized by low affinity.

Results of the cell viability highlighted the potential of the obtained complexes as promising candidates for further investigation, particularly for breast adenocarcinoma, while also emphasizing the importance of evaluating their safety in normal cell populations.

The effects of complex **2** on MDA and PC levels can vary significantly across different cancer cell types, potentially contributing to oxidative stress in breast adenocarcinoma while providing protective effects in pancreatic cancer cells. Overall, this indicates the complexity of ruthenium’s role in cancer therapy and underscores the importance of context-dependent responses in evaluating its therapeutic potential.

A reduction in MMP9 activity indicates that the complex **1** may be limiting the cancer cells’ invasive potential, thereby potentially hindering tumor progression. The statistical significance for breast and pancreatic cancer reinforces that these findings are unlikely due to random chance and highlights the treatment’s impact on modifying the tumor microenvironment. This outcome could be an essential marker of the complex **1** efficacy, providing a basis for further investigation into its therapeutic potential against these types of cancers.

The pattern of cell cycle dynamics indicates that complexes **1** and **2** exhibit different selectivity towards MIA PaCa-2 and MDA-MB-231 cancer cells. Complexes **1** and **2** may not exert the desired inhibitory effect on MDA-MB-231cells, potentially leading to continued growth and division of tumor cells. This lack of response observed in pancreatic cancer cells may suggest several possibilities; the complexes may not target the cellular pathways involved in cell cycle regulation, making them ineffective against the specific cancer or cell type being studied. Additionally, it could indicate that the cell type in question is resilient to the drug’s mechanisms, potentially due to existing resistance mechanisms or unique metabolic states. In some cases, the drug might predominantly affect other cellular processes, such as signaling pathways or apoptosis, without influencing cell cycle dynamics. These findings underline the importance of further investigations and evaluation of the specific mechanisms of action of **1** and **2**.

Comparative docking analysis of protein binding, focusing on key targets such as ACHE, MMP-9, and HSA, highlighted distinct binding profiles that underscore the importance of complex architecture in facilitating this interaction. Overall, the findings emphasize the potential for developing targeted ruthenium-based therapies, suggesting that mononuclear and binuclear complexes warrant further investigation for their roles in cancer treatment.

## Figures and Tables

**Table 1:** Binding contant (K_b_), the number of binding sites (n), ΔG_b_°, ΔH_b_°, and ΔS_b_° values for the **1/2**–HSA system at different temperatures.

Compelex	T [K]	K_b_[M^−1^]	ΔH_b_ [kJ mol^−1^]	ΔS_b_ [J mol^−1^ K^−1^]	ΔG_b_ [kJ mol^−1^]	n	R
**1**-HSA	300	9.50 × 10^−4^	149.8	590	−28.3	1.05	0.998
	305	1.75 × 10^−5^			−31.2	1.10	0.999
	310	6.53 × 10^−5^			−34.2	1.21	0.999
**2**-HSA	300	2.95 × 10^−4^	78.9	348	−25.6	1.00	0.998
	305	4.62 × 10^−4^			−27.4	1.03	0.995
	310	8.19 × 10^−4^			−29.1	1.09	0.996

**Table 2:** Contributions of the unbound 16-DS calculated from EPR spectral simulations for the HSA/16-DS samples in the absence and presence of Ru-complexes.

Sample ^1^	Unbound Component Contribution (X_UB_, %)
HSA/16-DS	0.50 ± 0.02
HSA/16-DS + 1.2% *v*/*v* DMSO	0.53 ± 0.01
HSA/16-DS/**1**	0.73 ± 0.02
HSA/16-DS/**2**	0.71 ± 0.02

^1^ All samples were prepared in the phosphate buffer (100 mM, pH 7.4) and contained 50 µM HSA and 250 µM 16-DS. The samples of Ru(II) complexes (200 µM) contained additionally 1.2% *v*/*v* DMSO. All the data are presented as an average of two replicates of the same sample.

**Table 3:** The calculated values of free energies of binding and inhibition constants of investigated compounds with ACHE, MMP-9, and BSA receptors.

	ΔG_bind_(kJ mol^−1^)	K_i_(µM)
**2**-ACHE	−36.3	4.3 × 10^−1^
**1**-ACHE	−39.6	1.1 × 10^−1^
**2**-MMP-9	−35.2	6.8 × 10^−1^
**1**-MMP-9	−41.5	5.3 × 10^−2^
**2**-HSA	−24.9	4.4 × 10^1^
**1**-HSA	−29.7	6.3

## Data Availability

The original contributions presented in the study are included in the article/Supplementary Materials, further inquiries can be directed to the corresponding author/s.

## Supplementary Materials

The following supporting information can be downloaded at: https://www.mdpi.com/article/10.3390/biom15030350/s1, Figure S1: ^1^H and ^13^C NMR spectra of **1** (DMSO-*d*_6_, 300 and 75 MHz, respectively); Figure S2: ^1^H and ^13^C NMR spectra of **2** (DMSO-*d*_6_, 300 and 75 MHz, respectively); Figure S3: UV-VIS spectra of 10^−4^ M solution in PBS of (a) complex **1** and (b) complex **2**, measured within 24 h after dissolution; Table S1: Experimental and theoretical (at B3LYP/6-311++G(d,p)(H,C,N,Cl)/def2-TZVP(Ru) level of theory) bond lengths (in Å); Table S2: Experimental and theoretical (at B3LYP/6-311++G(d,p)(H,C,N,Cl)/def2-TZVP(Ru) level of theory) bond angles (in °); Table S3: Selected bond lengths (Å) and angles (°) of the hydrogen bridges of **2b**; Table S4: Experimental, unscaled, and scaled theoretical (at B3LYP/6-311++G(d,p)(H,C,N,Cl)/def2-TZVP(Ru) level of theory) ^13^C NMR chemical shifts of **1** (in ppm); Table S5: Experimental, unscaled, and scaled theoretical (at B3LYP/6-311++G(d,p)(H,C,N,Cl)/def2-TZVP(Ru) level of theory) ^13^C NMR chemical shifts of **2** (in ppm); Table S6: The calculated Bond Critical Points (BCP) properties at the B3LYP/6-311++G(d,p)(H,C,N,Cl)/def2-TZVP(Ru) level of theory: the electron density (ρ(r)) and its Laplacian (∇^2^ρ(r)); the Lagrangian kinetic electron density (G(r)) and the potential electron density (V(r)); the density of the total energy of electrons (H(r))—Cremer-Kraka electronic energy density; the interatomic bond energy, E_bond_; Figure S4:The Bond Critical Points in the structures of complexes **1** and (**2**–Cl)**^+^**; Figure S5: The fluorescence emission spectra of HSA for the titration with various concentrations of complex **2** at (a) 27°, (b) 32°, and (c) 37 °C, and (d) the van ’t Hoff plot for the binding process; Figure S6: EPR spectra of HSA/16-DS at [HSA]:[16-DS] molar ratio 1:5 without Ru-complexes (a) and in the presence of Ru-complexes at the [HSA]:[Ru-complexes] molar ratio of 1:4–Ru-109 (b) and TE-101 (c). All samples were prepared in the phosphate buffer (100 mM, pH 7.4) and contained 50 µM HSA, 250 µM 16-DS, 200 µM Ru-complexes, and 1.2% *v*/*v* DMSO. The spectra were first baseline corrected, and normalized by dividing the whole dataset by the maxima of the central peak; Figure S7: Experimental and simulated EPR spectra of the HSA/16-DS in buffer ([HSA]:[16-DS] molar ratio was 1:5, concentrations of HSA and 16-DS were 50 µM and 250 µM, respectively). The latter three spectra represent strongly (SB), weakly bound (WB), and unbound (UB) components, respectively. All components, the total simulated and experimental spectra, are plotted on the same intensity scale.
