# Supplementary material for: Ru(II) Complexes with 3,4-Dimethylphenylhydrazine: Exploring In Vitro Anticancer Activity and Protein Affinities
Source: Biomolecules. 2025 Feb 28;15(3):350. doi: 10.3390/biom15030350 (PMC11940238; doi:10.3390/biom15030350)
Supplement: Supplementary file 1 [file biomolecules-15-00350-s001.zip › biomolecules-3476973-supplementary.pdf]

Ru(II) Complexes with 3,4-Dimethylphenylhydrazine: Exploring in vitro Anticancer Activity and Protein Affinities

Jasmina Dimitrić Marković[1]*, Dušan Dimić[1], Thomas Eichhorn[2], Dejan Milenković[3], Aleksandra Pavićević[1], Dragoslava Djikić[4], Emilija Živković[4], Vladan Čokić[4], Tobias Rüffer[5] and Goran N. Kaluđerović*[2]

[1] Faculty of Physical Chemistry University of Belgrade, Serbia; markovich@ffh.bg.ac.rs; ddimic@ffh.bg.ac.rs, aleks.pavicevic@ffh.bg.ac.rs
[2] Department of Engineering and Natural Sciences, University of Applied Sciences Merseburg, Germany; thomas.eichhorn@hs-merseburg.de; goran.kaluderovic@hs-merseburg.de
[3] Department of Science, Institute for Information Technologies, University of Kragujevac, Serbia; dejanm@uni.ac.kg
[4] Institute for Medical Research, University of Belgrade, Serbia; vl@imi.bg.ac.rs; dragoslava@imi.ac.bg.rs; emilija.zivkovic@imi.bg.ac.rs
[5] Institute of Chemistry, Chemnitz University of Technology, Straße der Nationen 62, D-09111 Chemnitz, Germany; tobias.rueffer@chemie.tu-chemnitz.de

*Correspondence: goran.kaluderovic@hs-merseburg.de (GK); markovich@ffh.bg.ac.rs (JDM);

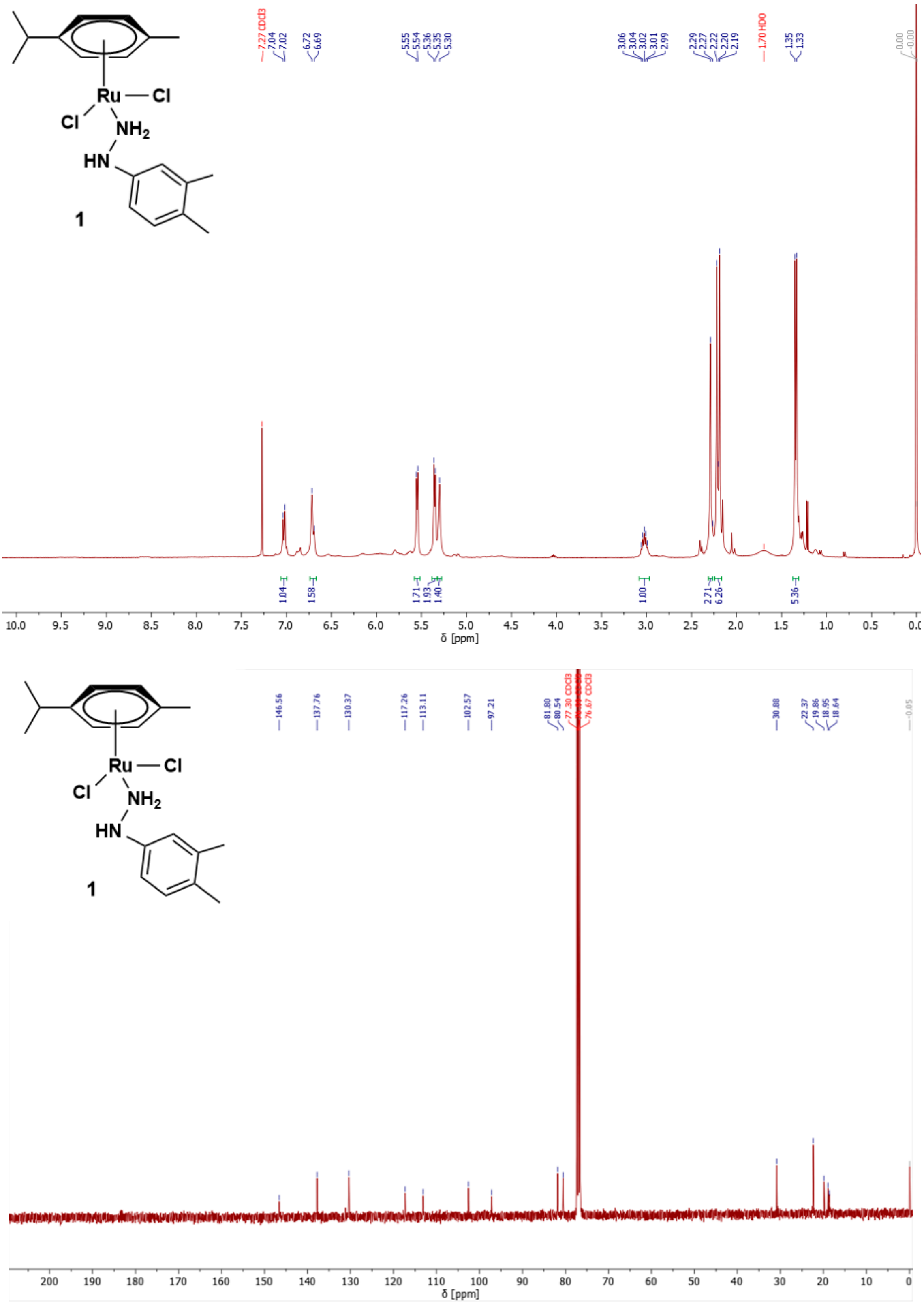

Figure S1. ^{1}H and ^{13}C NMR of **1** (DMSO-d_6, 300 and 75 MHz, respectively)

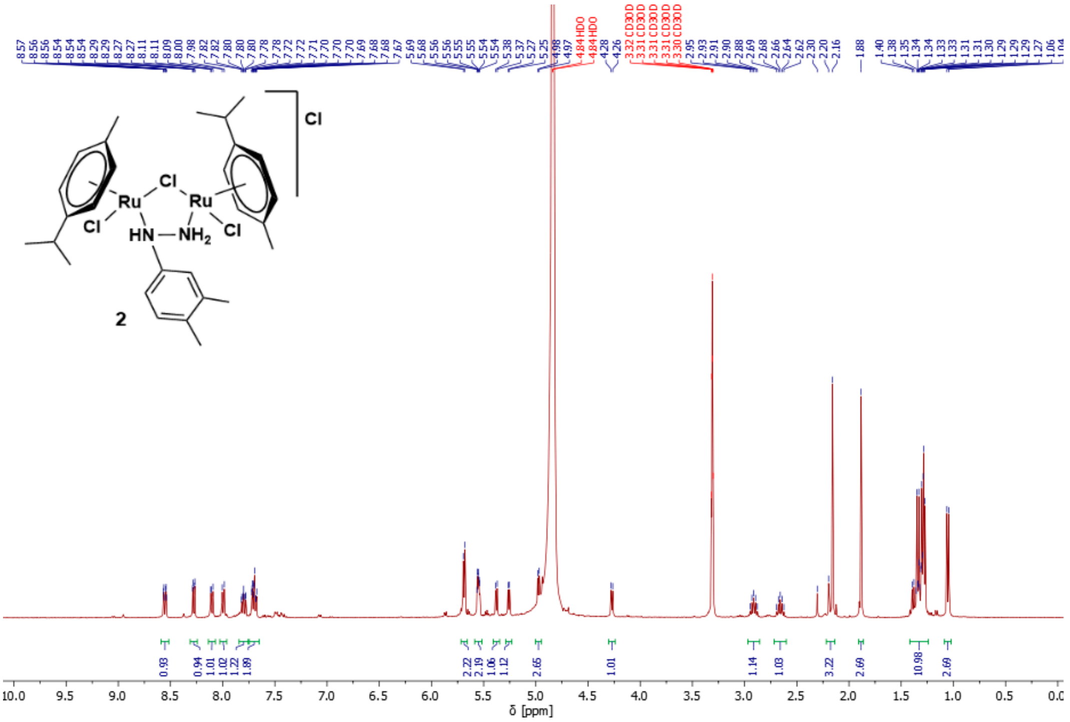

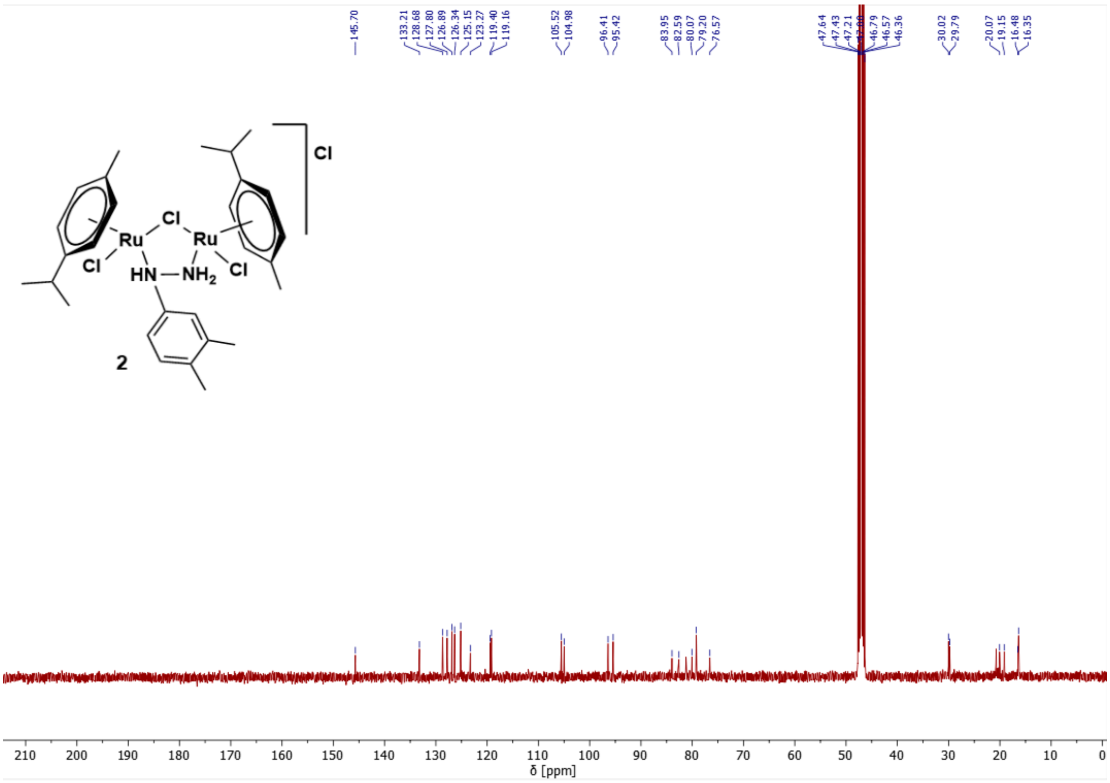

Figure S2. ^{1}H and ^{13}C NMR of **2** (DMSO-d_6, 300 and 75 MHz, respectively)

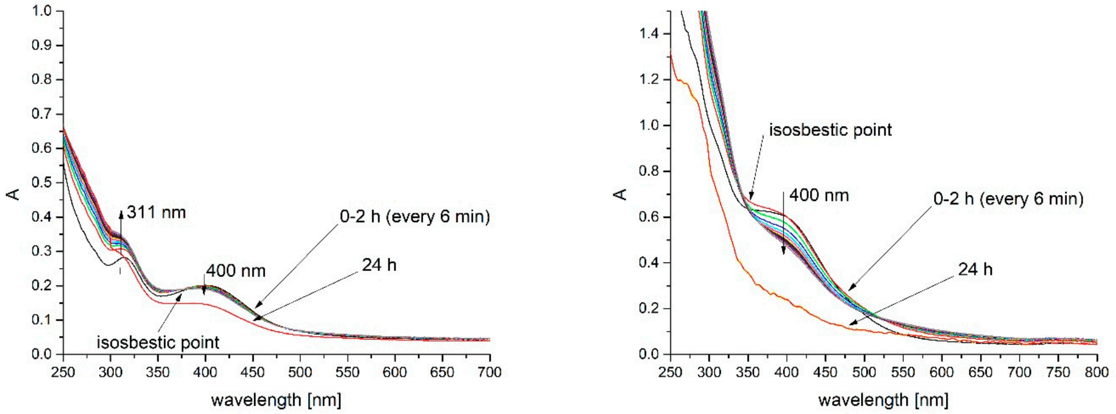

(a) (b)

Figure S3. UV-VIS spectra of 1×10^{-4} M solution in PBS of (a) complex **1** and (b) complex **2**, measured within 24 hours after dissolution

Table S1. Experimental and theoretical (at B3LYP/6-311++G(d,p)(H,C,N,Cl)/def2-TZVP(Ru) level of theory) bond lengths (in Å)

Bond	Experimental	Theoretical
C1-C2	1.391(9)	1.435
C1-C6	1.432(10)	1.413
C1-C7	1.515(10)	1.501
C1-Ru1	2.197(6)	2.225
C2-C3	1.421(8)	1.404
C2-Ru1	2.166(6)	2.188
C3-C4	1.406(8)	1.438
C3-Ru1	2.151(5)	2.200
C4-C5	1.450(8)	1.409
C4-C8	1.502(8)	1.517
C4-Ru1	2.195(6)	2.269
C5-C6	1.378(9)	1.431
C5-Ru1	2.173(6)	2.228
C6-Ru1	2.170(6)	2.190
C8-C10	1.503(9)	1.532
C8-C9	1.545(10)	1.545
C11-C12	1.405(9)	1.411
C11-C16	1.441(8)	1.436
C11-C17	1.495(9)	1.501
C11-Ru2	2.194(6)	2.232
C12-C13	1.416(9)	1.431
C12-Ru2	2.164(6)	2.198

C13-C14	1.414(8)	1.409
C13-Ru2	2.176(6)	2.224
C14-C15	1.416(9)	1.438
C14-C18	1.519(9)	1.517
C14-Ru2	2.206(6)	2.266
C15-C16	1.386(9)	1.404
C15-Ru2	2.170(5)	2.195
C16-Ru2	2.177(5)	2.188
C18-C19	1.524(10)	1.532
C18-C20	1.527(9)	1.545
C21-C22	1.39	1.394
C21-C26	1.39	1.392
C21-N2	1.530(7)	1.459
C22-C23	1.39	1.398
C23-C24	1.39	1.411
C23-C27	1.505(14)	1.508
C24-C25	1.39	1.399
C24-C28	1.533(12)	1.507
C25-C26	1.39	1.392
N1-N2	1.466(7)	1.471
N1-Ru1	2.136(5)	2.179
N2-Ru2	2.183(4)	2.220
Cl1-Ru1	2.4128(16)	2.438
Cl2-Ru2	2.4237(15)	2.472
Cl2-Ru1	2.4481(14)	2.478
Cl3-Ru2	2.4007(15)	2.441
R		0.999
MAE		0.025

Table S2. Experimental and theoretical (at B3LYP/6-311++G(d,p)(H,C,N,Cl)/def2-TZVP(Ru) level of theory) bond angles (in °)

Bond angle	Experimental	Theoretical
C2-C1-C6	117.5(6)	116.88
C2-C1-C7	122.8(7)	122.22
C6-C1-C7	119.6(6)	120.87
C2-C1-Ru1	70.2(3)	69.99
C6-C1-Ru1	69.8(4)	69.62
C7-C1-Ru1	128.1(5)	129.67
C1-C2-C3	121.4(6)	122.00
C1-C2-Ru1	72.6(4)	72.70

C3-C2-Ru1	70.2(3)	72.54
C4-C3-C2	121.6(5)	120.87
C4-C3-Ru1	72.8(3)	73.35
C2-C3-Ru1	71.4(3)	69.67
C3-C4-C5	116.5(5)	117.28
C3-C4-C8	118.3(5)	119.10
C5-C4-C8	125.1(6)	123.62
C3-C4-Ru1	69.4(3)	70.16
C5-C4-Ru1	69.8(3)	68.63
C8-C4-Ru1	132.2(4)	132.31
C6-C5-C4	121.2(6)	121.66
C6-C5-Ru1	71.4(4)	70.88
C4-C5-Ru1	71.4(3)	73.87
C5-C6-C1	121.8(6)	121.24
C5-C6-Ru1	71.6(3)	71.79
C1-C6-Ru1	71.9(4)	72.45
C4-C8-C10	114.9(6)	114.68
C4-C8-C9	107.8(6)	108.43
C10-C8-C9	111.0(6)	111.14
C12-C11-C16	117.1(6)	117.08
C12-C11-C17	121.6(6)	122.12
C16-C11-C17	121.3(6)	120.78
C12-C11-Ru2	70.0(3)	70.12
C16-C11-Ru2	70.1(3)	69.40
C17-C11-Ru2	128.5(4)	129.92
C11-C12-C13	121.9(5)	121.81
C11-C12-Ru2	72.4(3)	72.74
C13-C12-Ru2	71.4(3)	72.11
C14-C13-C12	120.7(6)	121.12
C14-C13-Ru2	72.3(3)	73.33
C12-C13-Ru2	70.5(4)	70.13
C13-C14-C15	117.3(6)	117.06
C13-C14-C18	122.5(6)	123.80
C15-C14-C18	120.2(5)	119.14
C13-C14-Ru2	70.0(3)	70.11
C15-C14-Ru2	69.8(3)	68.53
C18-C14-Ru2	130.6(4)	132.33
C16-C15-C14	122.4(5)	121.80
C16-C15-Ru2	71.7(3)	71.06
C14-C15-Ru2	72.5(3)	73.89
C15-C16-C11	120.6(6)	121.06
C15-C16-Ru2	71.2(3)	71.57
C11-C16-Ru2	71.4(3)	72.70
C14-C18-C20	107.6(6)	108.44

C19-C18-C20	110.8(6)	114.70
C22-C21-C26	120	120.46
C22-C21N2	112.5(6)	118.61
C26-C21-N2	127.4(6)	120.93
C23-C22-C21	120	121.01
C24-C23-C22	120	119.02
C24-C23-C27	121.0(7)	121.06
C22-C23-C27	119.0(7)	119.91
C23-C24-C25	120	118.84
C23-C24-C28	121.2(7)	120.93
C25-C24-C28	118.8(7)	120.22
C26-C25-C24	120	122.10
C25-C26-C21	120	118.56
N2-N1-Ru1	115.0(3)	115.87
N1-N2-C21	105.4(5)	111.69
N1-N2-Ru2	113.7(3)	111.85
C21-N2-Ru2	111.3(4)	118.01
Ru2-Cl2-Ru1	109.59(5)	110.73
N1-Ru1-C3	91.2(2)	97.28
N1-Ru1-C2	93.7(2)	94.45
C3-Ru1-C2	38.4(2)	37.80
N1-Ru1-C6	158.5(2)	154.47
C3-Ru1-C6	80.5(2)	79.42
C2-Ru1-C6	67.6(2)	67.32
N1-Ru1-C5	154.0(2)	160.62
C3-Ru1-C5	68.3(2)	66.59
C2-Ru1-C5	80.3(2)	79.35
C6-Ru1-C5	37.0(2)	37.33
N1-Ru1-C4	115.54(19)	123.21
C3-Ru1-C4	37.7(2)	36.49
C2-Ru1-C4	68.9(2)	67.25
C6-Ru1-C4	68.7(2)	67.64
C5-Ru1-C4	38.8(2)	37.50
N1-Ru1-C1	120.3(2)	117.29
C3-Ru1-C1	68.6(2)	67.92
C2-Ru1-C1	37.2(2)	37.31
C6-Ru1-C1	38.3(3)	37.94
C5-Ru1-C1	68.3(2)	67.98
C4-Ru1-C1	82.0(2)	80.46
N1-Ru1-Cl1	82.90(15)	84.23
C3-Ru1-Cl1	120.71(17)	120.32
C2-Ru1-Cl1	159.00(17)	158.06
C6-Ru1-Cl1	118.4(2)	114.81
C5-Ru1-Cl1	93.68(17)	89.81

C4-Ru1-Cl1	93.82(17)	91.85
C1-Ru1-Cl1	156.00(19)	152.05
N1-Ru1-Cl2	85.02(12)	82.38
C3-Ru1-Cl2	151.75(17)	151.12
C2-Ru1-Cl2	113.76(17)	113.87
C6-Ru1-Cl2	92.89(17)	94.17
C5-Ru1-Cl2	120.60(16)	122.31
C4-Ru1-Cl2	159.35(16)	160.23
C1-Ru1-Cl2	89.14(17)	90.43
Cl1-Ru1-Cl2	86.67(5)	88.05
C12-Ru2-C15	80.0(2)	79.32
C12-Ru2-C13	38.1(2)	37.76
C15-Ru2-C13	67.6(2)	66.66
C12-Ru2-C16	68.0(2)	67.24
C15-Ru2-C16	37.2(2)	37.37
C13-Ru2-C16	80.4(2)	79.46
C12-Ru2-N2	95.4(2)	95.82
C15-Ru2-N2	152.9(2)	157.56
C13-Ru2-N2	92.3(2)	96.04
C16-Ru2-N2	160.9(2)	157.77
C12-Ru2-C11	37.6(2)	37.14
C15-Ru2-C11	68.5(2)	67.91
C13-Ru2-C11	68.7(2)	67.75
C16-Ru2-C11	38.5(2)	37.90
N2-Ru2-C11	122.4(2)	120.26
C12-Ru2-C14	68.5(2)	67.27
C15-Ru2-C14	37.8(2)	37.58
C13-Ru2-C14	37.6(2)	36.56
C16-Ru2-C14	68.2(2)	67.76
N2-Ru2-C14	115.7(2)	120.36
C11-Ru2-C14	81.8(2)	80.39
C12-Ru2-Cl3	114.99(18)	118.53
C15-Ru2-Cl3	119.64(18)	116.48
C13-Ru2-Cl3	152.85(18)	156.29
C16-Ru2-Cl3	91.90(18)	90.44
N2-Ru2-Cl3	86.68(15)	85.21
C11-Ru2-Cl3	89.07(18)	91.12
C14-Ru2-Cl3	157.39(16)	153.95
C12-Ru2-Cl2	157.07(18)	153.21
C15-Ru2-Cl2	91.87(17)	92.03
C13-Ru2-Cl2	119.05(18)	115.60
C16-Ru2-Cl2	116.94(17)	119.18
N2-Ru2-Cl2	82.07(14)	82.52
C11-Ru2-Cl2	155.08(15)	157.07

C14-Ru2-Cl2	91.98(16)	90.40
R		0.998
MAE [°]		1.50

Table S3. Selected bond lengths (Å) and angles (°) of the hydrogen bridges of **2b**.

D–H···A	D···A	D–H···A
N1–H1N···Cl6	3.228(5)	167(5)
N1–H2N···Cl3	3.032(5)	136(5)
N1–H2N···Cl5	3.219(5)	122(5)
N2–H3N···Cl1	3.042(6)	144(5)

Table S4. Experimental, unscaled, and scaled theoretical (at B3LYP/6-311++G(d,p)(H,C,N,Cl)/def2-TZVP(Ru) level of theory) ^{13}C NMR chemical shifts of **1** (in ppm)

C-atom	Experimental	Theoretical	
		Unscaled	Scaled
C*C*H_{3cym}	18.7	20.2	18.9
C*C*H3	19	22.1	20.7
C*C*H3	19.9	23.0	21.6
CH(*C*H_3)$_2$	22.4	24.0	22.4
*C*H(CH_3)$_2$	30.9	37.4	35.0
C*C*HCH$_{cym}$	80.6	87.7	82.2
C*C*HCH$_{cym}$	81.8	88.3	82.7
CH_3*C*$_{cym}$	97.3	104.5	97.9
*C*CH(CH_3)$_2$	102.6	112.4	105.2
CH*C*HCN$_{Ph}$	113.1	118.2	110.6
C*C*HCN$_{Ph}$	117.3	126.0	118.0
*C*HCHCN$_{Ph}$	130.4	137.4	128.7
*C*CCHCN$_{Ph}$	131.2	140.2	131.3
C*C*CHCN$_{Ph}$	137.8	148.8	139.4
CN$_{Ph}$	146.6	152.8	143.0
R		0.999	0.999
MAE [ppm]		6.22	1.55

Table S5. Experimental, unscaled, and scaled theoretical (at B3LYP/6-311++G(d,p)(H,C,N,Cl)/def2-TZVP(Ru) level of theory) ^{13}C NMR chemical shifts of **2** (in ppm)

C-atom	Experimental	Theoretical	
		Unscaled	Scaled
C*C*H_{3cym}	16.3	19.8	17.9

C*C*H$_{3cym}$	16.5	20.4	18.5
C*C*H$_3$	19.1	22.6	20.5
C*C*H$_3$	20.1	23.0	20.8
CH(*C*H$_3$)$_2$	29.8	23.8	21.6
CH(*C*H$_3$)$_2$	30	37.5	33.9
C*C*HCH$_{cym}$	76.6	84.0	76.0
C*C*HCH$_{cym}$	79.2	86.8	78.6
CCH*C*H$_{cym}$	80.1	93.3	84.5
C*C*HCH$_{cym}$	82.6	90.3	81.8
CCH*C*H$_{cym}$	83.9	93.9	85.0
CH$_3$*C*$_{cym}$	95.4	101.1	91.5
CH3*C*$_{cym}$	96.4	107.6	97.4
*C*CH(CH$_3$)$_2$	105	117.3	106.1
*C*CH(CH$_3$)$_2$	105.5	121.9	110.4
CH*C*HCN$_{Ph}$	119.2	126.9	114.9
CC*C*HCN$_{Ph}$	123.3	127.2	115.1
*C*HCHCN$_{Ph}$	126.9	139.2	126.0
C*C*CHCN$_{Ph}$	128.7	148.8	134.7
C*C*CHCN$_{Ph}$	133.2	149.1	135.0
*C*N$_{Ph}$	145.7	157.5	142.6
R		0.992	0.992
MAE [ppm]		9.08	2.88

Table S6. The calculated Bond Critical Points (BCP) properties at the B3LYP/6-311++G(d,p)(H,C,N,Cl)/def2-TZVP(Ru) level of theory: the electron density (ρ(r)) and its Laplacian (∇^2ρ(r)); the Lagrangian kinetic electron density (G(r)) and the potential electron density (V(r)); the density of the total energy of electrons (H(r)) – Cremer-Kraka electronic energy density; the interatomic bond energy, E_{bond},

Bond	ρ(r) [a.u.]	∇^2ρ(r) [a.u.]	G(r) [kJ mol^{-1}]	V(r) [kJ mol^{-1}]	H(r) [kJ mol^{-1}]	-G(r)/V(r)	E_{bond} [kJ mol^{-1}]
Complex **1**							
Ru-C1	0.083	0.239	210.3	-263.3	-53.0	0.8	-131.6
Ru-C2	0.084	0.241	211.1	-266.1	-55.0	0.8	-133.0
Ru-C3	0.081	0.243	207.2	-255.5	-48.2	0.8	-127.7
Ru-C4	0.083	0.240	209.4	-261.7	-52.4	0.8	-130.9
Ru-Cl1	0.066	0.185	152.1	-183.5	-31.4	0.8	-91.8
Ru-Cl2	0.068	0.185	234.9	-273.5	-38.6	0.9	-136.8
Ru-N	0.082	0.302	154.3	-187.6	-33.3	0.8	-93.8
Cl···H−C	0.005	0.015	8.1	-6.2	1.9	1.3	-3.1
Cl···H−C	0.008	0.026	13.7	-10.5	3.2	1.3	-5.2
C···H−C	0.005	0.016	8.4	-6.5	1.9	1.3	-3.2
C···H−C	0.002	0.006	3.2	-2.4	0.8	1.3	-1.2
Cl···H−N	0.17	0054	30.4	-25.6	4.8	1.2	-12.8
Complex **2**							

Ru1-C1	0.085	0.232	208.7	-265.7	-57.0	0.8	-132.9
Ru1-C2	0.084	0.230	206.0	-261.5	-55.5	0.8	-130.8
Ru1-C3	0.084	0.234	207.7	-262.8	-55.1	0.8	-131.4
Ru1-Cl1	0.059	0.190	147.2	-170.3	-23.1	0.9	-85.2
Ru1-Cl2	0.066	0.188	154.2	-185.4	-31.2	0.8	-92.7
Ru1-N	0.073	0.289	216.9	-245.3	-28.4	0.9	-122.7
Ru2-C1	0.082	0.238	208.2	-260.8	-52.6	0.8	-130.4
Ru2-C2	0.086	0.230	207.8	-265.2	-57.4	0.8	-132.6
Ru2-C3	0.086	0.228	207.5	-265.8	-58.3	0.8	-132.9
Ru2-Cl1	0.067	0.189	154.5	-185.9	-31.4	0.8	-92.9
Ru2-Cl2	0.060	0.191	148.4	-172.3	-23.9	0.9	-86.1
Ru2-N	0.080	0.318	242.5	-278.2	-35.7	0.9	-139.1
Cl···H−C	0.006	0.017	8.9	-6.8	2.0	1.3	-3.4
Cl···H−C	0.028	0.077	51.1	-43.5	7.6	1.2	-21.7
Cl···H−C	0.006	0.018	9.8	-7.5	2.2	1.3	-3.8
Cl···H−C	0.007	0.024	12.8	-9.6	3.2	1.3	-4.8
Cl···H−C	0.003	0.077	50.0	-49.8	0.2	1.0	-24.9
Cl···H−C	0.008	0.025	13.5	-10.6	2.9	1.3	-5.3
H···C−H	0.002	0.008	4.0	-2.8	1.2	1.4	-1.4

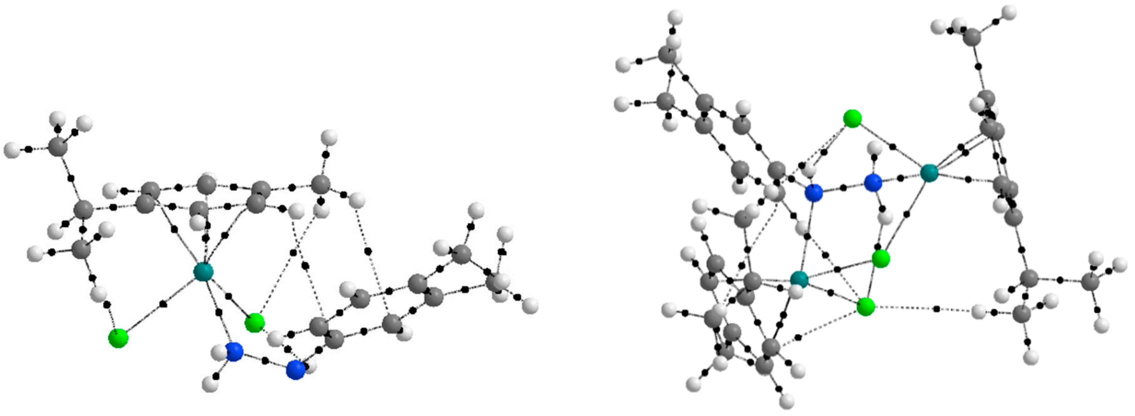

(a) (b)

Figure S4. The Bond Critical Points in the structures of complexes **1** and $\mathbf{2}^{+}$.

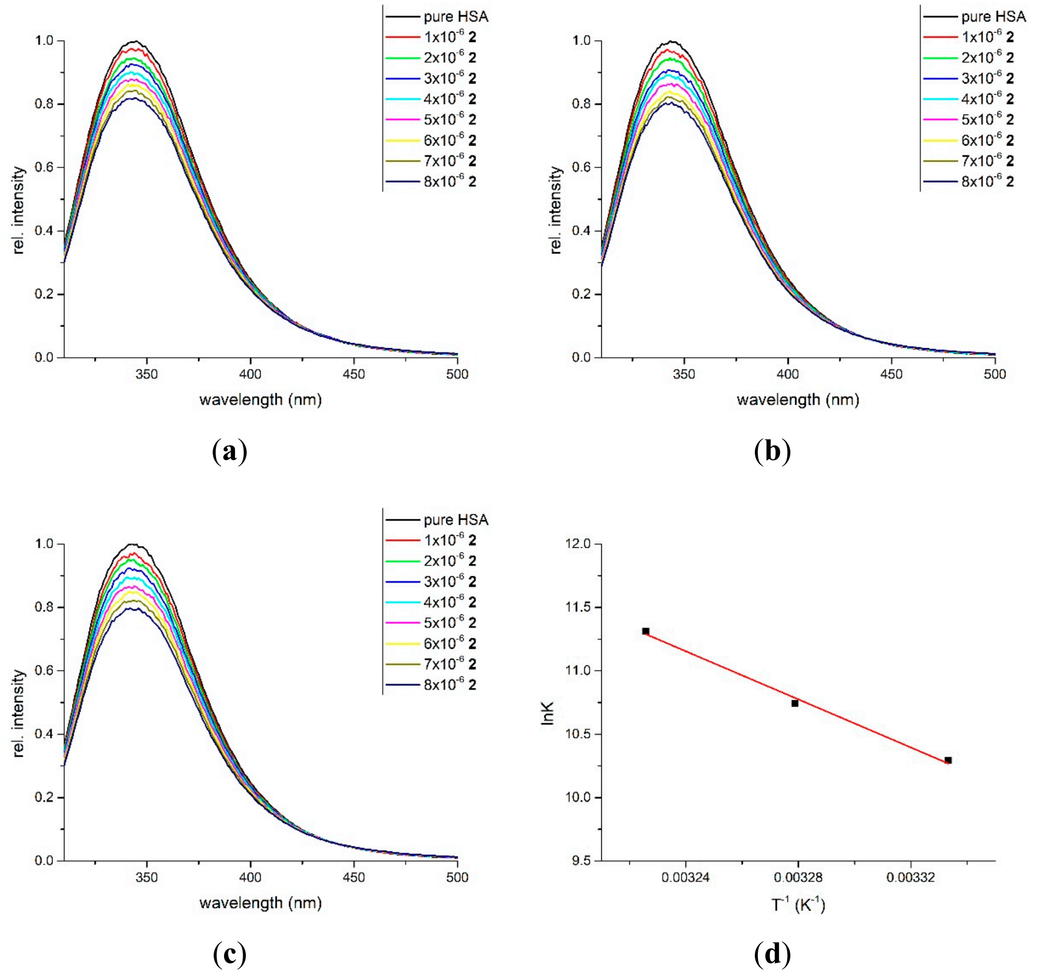

Figure S5. The fluorescence emission spectra of HSA for the titration with various concentrations of complex **2** at (a) 27°, (b) 32°, and (c) 37 °C, and (d) the van ’t Hoff plot for the binding process.

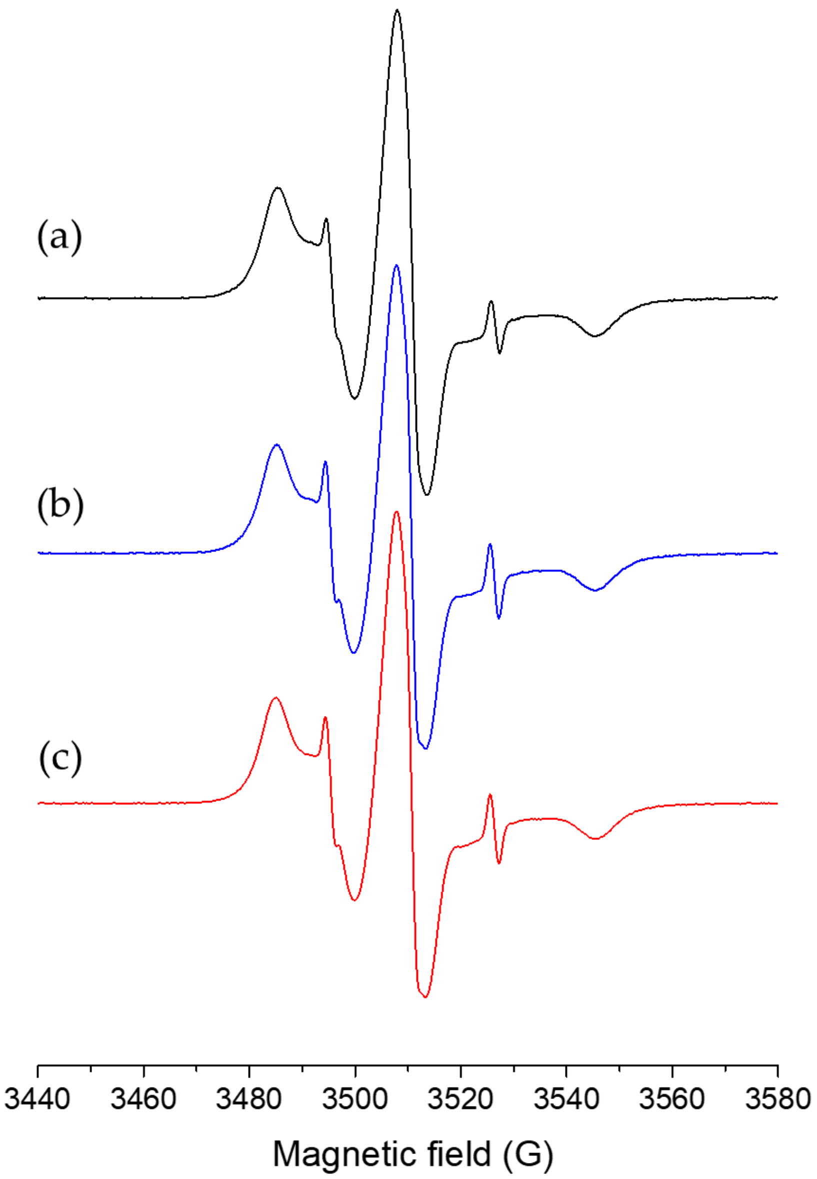

Figure S6. EPR spectra of HSA/16-DS at [HSA]:[16-DS] molar ratio 1:5 without Ru-complexes (a) and in the presence of Ru-complexes at the [HSA]:[Ru-complexes] molar ratio of 1:4 – compound 1 (b) and compound 2 (c). All samples were prepared in the phosphate buffer (100 mM, pH 7.4), and contained 50 µM HSA, 250 µM 16-DS, 200 µM Ru-complexes, and 1.2 % v/v DMSO. The spectra were first baseline corrected, and normalized by dividing the whole dataset by the maxima of the central peak.

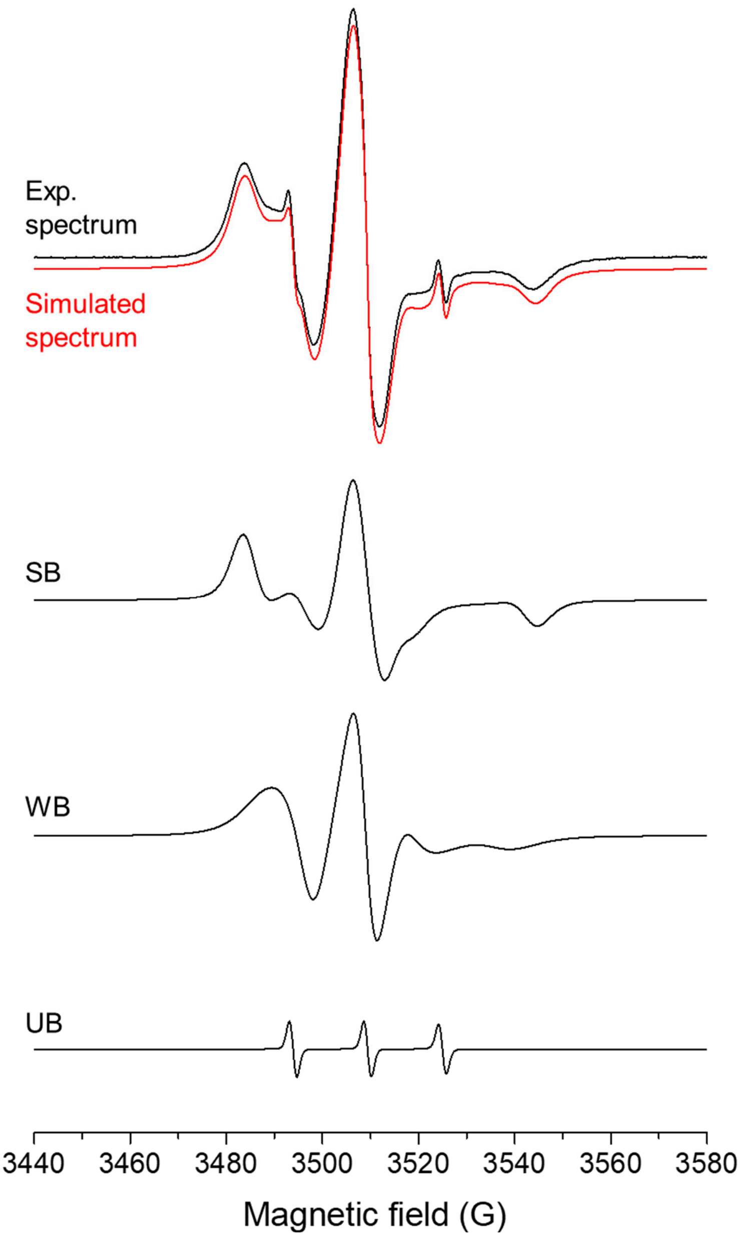

figure s7. experimental and simulated epr spectra of the hsa/16-ds in buffer ([hsa]:[16-ds] molar ratio was 1:5, concentrations of HSA and 16-DS were 50 µM and 250 µM, respectively). The latter three spectra represent strongly (SB), weakly bound (WB) and unbound (UB) component, respectively. All components, the total simulated and experimental spectra are plotted on the same intesity scale.
